# Current Knowledge on Spinal Meningiomas Epidemiology, Tumor Characteristics and Non-Surgical Treatment Options: A Systematic Review and Pooled Analysis (Part 1)

**DOI:** 10.3390/cancers14246251

**Published:** 2022-12-19

**Authors:** Victor Gabriel El-Hajj, Jenny Pettersson-Segerlind, Alexander Fletcher-Sandersjöö, Erik Edström, Adrian Elmi-Terander

**Affiliations:** 1 Department of Clinical Neuroscience, Karolinska Institutet, 171 77 Stockholm, Sweden; 2 Stockholm Spine Center, Löwenströmska Hospital, 194 45 Upplands-Väsby, Sweden

**Keywords:** spinal meningiomas, epidemiology, tumor characteristics, WHO grade, non-surgical treatment options, radiation

## Abstract

**Simple Summary:**

Spinal meningiomas are the most common adult primary intradural spinal tumors and an up-to-date comprehensive summary of the current knowledge is greatly needed. An extensive review of the literature covering all aspects of spinal meningiomas was performed and a total of 104 studies were included. The pooled analysis revealed that most patients were female, in their seventh decade of life, with WHO grade 1 tumors. Moreover, interregional and age-related differences in epidemiology and/or histology were found. Sensory and motor dysfunction as well as pain were the most common presenting symptoms. Despite some promising results, the benefits of non-surgical treatments remain controversial.

**Abstract:**

Background: Spinal meningiomas are the most common primary intradural spinal tumors. Although they are a separate entity, a large portion of the knowledge on spinal meningiomas is based on findings in intracranial meningiomas. Therefore, a comprehensive review of all the literature on spinal meningiomas was performed. Methods: Electronic databases were searched for all studies on spinal meningiomas dating from 2000 and onward. Findings of matching studies were pooled to strengthen the current body of evidence. Results: A total of 104 studies were included. The majority of patients were female (72.83%), elderly (peak decade: seventh), and had a world health organization (WHO) grade 1 tumor (95.7%). Interestingly, the minority of pediatric patients had a male overrepresentation (62.0% vs. 27.17%) and higher-grade tumors (33.3% vs. 4.3%). Sensory and motor dysfunction and pain were the most common presenting symptoms. Despite a handful of studies reporting promising findings associated with the use of non-surgical treatment options, the literature still suffers from contradictory results and limitations of study designs. Conclusions: Elderly females with WHO grade 1 tumors constituted the stereotypical type of patient. Compared to surgical alternatives, the evidence for the use of non-surgical treatments is still relatively weak.

## 1. Introduction

Meningiomas arise from arachnoid cap cells in the leptomeninges surrounding the brain or spinal cord. Although frequent among intradural tumors, spinal meningiomas only make up a fraction of all meningiomas, as they are 10 to 50 times less prevalent than their intracranial counterparts [1,2,3]. The majority of spinal meningiomas are benign WHO grade 1 tumors [4,5,6,7] that primarily affect elderly women [4,6]. Most of these tumors are sporadic, but there is a known genetic link to neurofibromatosis type 2 (NF2), and exposure to ionizing radiation is a known predisposing factor [1,8]. The presence of estrogen or progesterone receptors in meningiomas [9] and the well-established ties to oral contraceptive drugs [10] has led to the hypothesis postulating pregnancy as a risk factor for the tumor [11,12]. However, a large population-based cohort study did not find support for this association [13]. These tumors can cause neurological impairments and pain due to local compression of the spinal cord and intradural structures [4,14]. Meningiomas are typically diagnosed on MRI, where they present with homogenous contrast enhancement and a dural tail, a thickening of the peritumoral dura [15]. Based on data from the management of intracranial meningiomas [16], adjuvant or salvage therapy is often advocated for recurrent or higher WHO grade tumors (WHO 2–3) and for patients who are unfit for surgery [17,18]. However, the effect and benefit of alternative treatments in the management of spinal meningiomas is unclear.

Many questions regarding the epidemiology, histopathology, clinical presentation, and diagnostics of spinal meningiomas remain unanswered. This systematic review aims to provide a comprehensive overview of the current knowledge on spinal meningiomas by summarizing, analyzing, and pooling published data. The results may serve as a knowledge base and act as a foundation for treatment decisions. The review also intends to identify knowledge gaps to guide future research efforts. This review will follow the outlines proposed in a previously published protocol [19].

## 2. Materials and Methods

This systematic review was performed in accordance with the Preferred Reporting Items for Systematic Reviews and Meta-Analyses (PRISMA) guidelines [20]; the related 2020-PRISMA checklist is provided as supplementary material (Supplementary File S1). The review protocol was registered within the International Prospective Register of Systematic Reviews (PROSPERO) (Registration ID: CRD42022330809 and Date of registration: 17 May 2022). The record was consistently updated in the event of any major change to the design of the work and the study protocol has previously been published [19].

### 2.1. Eligibility Criteria

Briefly, the inclusion criteria for this systematic review were peer-reviewed studies with at least ten patients, published after 2000, in the English language, covering the field of spinal meningiomas (Table 1). A control group was not deemed necessary for inclusion. Case reports, reviews, editorials, letters, and conference abstracts were excluded.

### 2.2. Databases and Search Strategy

An electronic database search was performed in PubMed, Embase, and Web of Science. The search was based on the keywords “spinal” and “meningioma”. Appropriate filters were used to exclude unsolicited publication types, non-English studies, and those published prior to the year 2000 (Supplementary File S2). The reference lists of the included studies were also searched to include studies otherwise overlooked.

### 2.3. Study Selection

**Figure 1:**
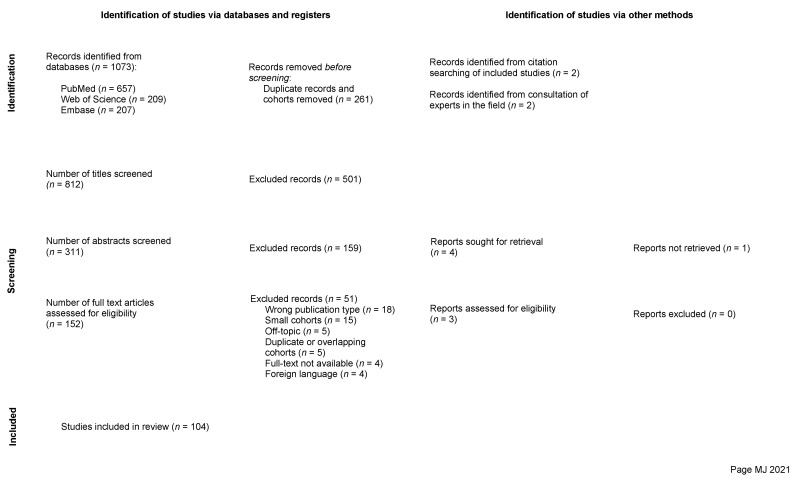
PRISMA flow chart illustrating the study selection process.

The searches yielded a total of 1073 papers, which was reduced to 807 after removal of duplicates. The final list of articles was uploaded to Rayyan [21] where the reviewing process took place. At first, the studies were screened by title and then abstract in subsequent steps by one of the reviewers (V.G.E.-H.). The remaining 147 articles were thereafter screened in full-text form and assessed by three independent and blinded reviewers (V.G.E.-H., A.E.-T. and E.E.). The final screening process resulted in 104 studies that were included. Inter-reviewer conflicts were resolved through discussion and subsequent unanimous decision. The reasons for exclusion in this last step were: wrong publication type (*n* = 18), small cohorts less than 10 cases (*n* = 15), off-topic (*n* = 5), duplicate or overlapping cohorts (*n* = 5), full-text not available (*n* = 4), and foreign language (*n* = 4). The selection process is illustrated in the PRISMA flowchart presented (Figure 1), and the final selection of studies included in this review is provided separately (Supplementary File S3, Table S1).

### 2.4. Data Extraction

Data from selected records were collected using a predefined extraction template, including (1) general publication information, (2) patient characteristics and epidemiology, (3) intervention characteristics, (4) study characteristics, and (5) outcomes.

### 2.5. Individual Evidence Level and Risk of Bias Assessment

To rate the research design quality, the studies were grouped according to evidence level, adhering to the modified Oxford Center for Evidence-Based Medicine (OCEBM) system [22]. Most studies provided level II evidence. To evaluate the risk of bias (ROB) among included studies, the Newcastle-Ottawa Scale (NOS) was used [23]. As most studies were single-armed, the NOS was modified to exclude modules intended for two-arm studies with comparator or control groups. Consequently, the scale allowed for a maximum of six points (instead of nine) to be allocated for each of the studies. Most studies were found to have a low overall risk of bias. An individual quality score (IQS) was then calculated for each of the studies based on both OCEBM and NOS grades. The detailed assessments are provided in the supplementary material (Supplementary File S3, Table S2).

### 2.6. Data Synthesis, Analysis, and Statistics

The heterogeneity among studies including methodological and design differences precluded a meta-analysis of the data. Instead, pooled analysis or qualitative synthesis were preferred as per the Cochrane recommendations [24]. Pooling was carried out by merging the data from different studies to create weighted averages. The weights considered were the sample sizes. Additionally, qualitative synthesis relied on the presentation of associations as established in each of the studies without processing or acting upon the data itself in any way.

When the primary intention of the analysis was to convey generalizable results in different contexts such as epidemiology or WHO grade distribution, special care was taken to select only studies that were representative for the population of patients with spinal meningiomas. In other words, studies with no inclusion biases towards the subjects studied, to avoid selection bias and preserve the external validity of the results. In some instances, studies with overlapping or identical cohorts were kept, as they provided different information of interest to this review. In such cases, either sample size or relevancy of the study determined which of the studies was regarded. In case missing pieces of information within studies were deemed valuable enough to the integrity of our results, the corresponding authors were contacted and asked to complete the data as needed. In this review, all statistical analyses and graphs were performed using the R software [25].

## 3. Results and Discussion

### 3.1. Epidemiology

Sixty-six representative studies including 25,275 patients were considered in this section (Supplementary File S3, Table S3).

#### 3.1.1. Sex

Sixty of the studies presented data on the male to female distribution, and of the 25,008 patients included, most were female (72.83%). The corresponding male to female ratio was 1:2.7, (range 1:1.5 to 1:14.5). The highest and lowest ratios were found in studies with relatively small sample sizes.

#### 3.1.2. Age

Adjusted for sample size, the average age of patients with spinal meningiomas across all representative studies (53 studies with 10,759 patients) was 62.6 years. Among these studies, six had limited their cohorts to adult patients only. These studies were kept as they only dismissed pediatric cases, which constitute a very small part of the population of spinal meningiomas. Excluding these studies from the pooled analysis resulted in a drop in the average age by 1.4 years.

According to seven studies on 16,672 patients, the proportion of pediatric (≤18 years), adult (19–69 years) and elderly (≥70 years) patients ranged between 0–2.5%, 61–90%, and 10–38%, respectively. Information on peak incidence was available in 19 studies with a total of 5627 patients. The peak incidence occurred between the seventh and eighth decades of life, with the seventh being the most frequently reported.

Average age in males vs. females was reported in 14 studies with a total of 1254 patients. Eight studies concluded that males were on average younger at the time of diagnosis, while six studies reported either the opposite or no significant difference. Three studies including 162 elderly patients (≥70) reported a total of 132 females, with a male to female ratio of 1:4.4, which differs considerably from the previously calculated ratio for the whole meningioma population (1:2.7) [26,27,28]. While the adult and elderly patients tended to be females to a higher extent, three studies including a total of 368 pediatric cases (≤18) showed a clear preponderance of males (62.0%) [29,30,31].

#### 3.1.3. Racial Distribution and Interregional Variability

The racial distribution of cases was mentioned in three studies including 17,940 patients [3,29,32]. All three studies consistently showed a preponderance of White patients (13,862; 77.3%) compared to other races. The authors provided no explanation as to the origin of such differences. Since all three studies originated from the United States, these results are not reflective of the global incidence of spinal meningiomas in different populations, but rather of the distribution among care-seeking patients in the USA.

Interregional comparisons revealed that the female predominance was more pronounced in Europe and East Asia compared to the United States, and that patients were on average approximatively seven and ten years younger in East Asia compared to the United States and Europe, respectively.

In summary, a clear female preponderance was seen (1:2.7) and the peak incidence falls within the seventh decade of life. Male patients seem to be younger on average and hence occupy a larger proportion of pediatric and young-adult cohorts, while elderly patients tend to be female. An interregional variability regarding both age and sex distribution seems to be present.

### 3.2. Histopathology

There were 65 studies reporting WHO grades of the surgically-treated tumors. Among these, 55 studies with a total of 5641 tumors were sufficiently representative of the population of patients with spinal meningiomas (Supplementary File S3, Table S4). The rest of the studies were either limited to specific age ranges (*n* = 4) or WHO grades (*n* = 6). Studies exclusively targeting a unique WHO grade were considered in their respective sections. Classifications are based on the 2016 WHO Classification of Tumors of the Central Nervous System [33].

#### 3.2.1. WHO Grade 1

**Figure 2:**
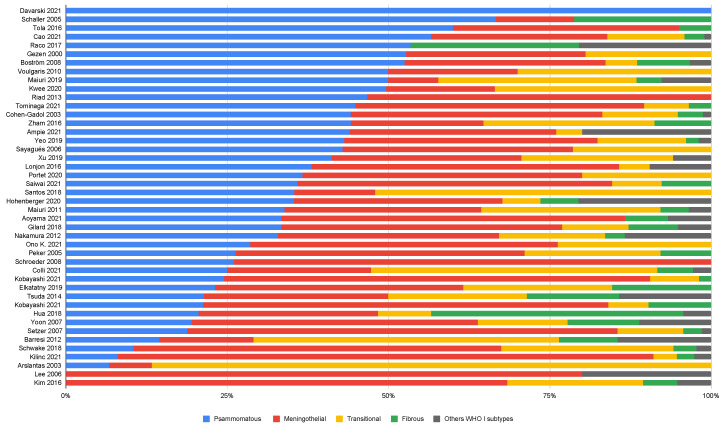
Distribution of the four most common meningioma subtypes as reported by the studies.

In the pooled sample of 5641 tumors, 5329 tumors were WHO grade 1 (95.5%). Based on 44 studies including 3859 tumors with histopathological subtyping, four of the nine described subtypes were consistently predominant (Figure 2). These were the Psammomatous (*n* = 1549, 40.1%,), Meningothelial (*n* = 1312, 34.0%), Transitional (*n* = 537, 13.9%) and Fibrous subtypes (*n* = 331, 8.6%). Altogether, the rarer subtypes accounted for about 2% of the total, with Angiomatous (*n* = 42) and Metaplastic spinal meningiomas (*n* = 27) being the most common, and Lymphoplasmacyte-rich (*n* = 4), Microcystic (*n* = 4), and Secretory (*n* = 2) being rarely reported.

In summary, the most common histological subtype across studies was any of the four dominating subtypes, i.e., Psammomatous, Meningothelial, Transitional, and Fibrous. The pooled analysis revealed that this order was the same as the frequency order of these subtypes.

#### 3.2.2. WHO Grade 2 and 3

There were 243 WHO grade 2 spinal meningiomas diagnosed among the 5641 tumors reported (4.3%). Histological subtyping was available in 191 of these tumors. An additional 40 tumors were identified in studies specifically addressing WHO grade 2 spinal meningiomas, bringing the total to 231. Of these, 154 (66.7%) were Atypical, 68 (29.4%) were Clear cell, and nine (3.9%) were Chordoid. This distribution was uniform across all studies.

A very limited number of WHO grade 3 spinal meningiomas have been reported in the literature. In the representative sample of 5641 tumors, only 24 were identified. Thus, the WHO grade 3 tumors constitute 0.4% of the total, highlighting the scarcity of this malignant entity. Of these 24 tumors, the histological subtype was available for 19; an additional five tumors with known histological subtypes were added from the non-representative studies. The Anaplastic subtype was the most prevalent with 17 (71%) tumors, while seven (29%) tumors were of the Papillary subtype. No Rhabdoid spinal meningiomas were reported.

#### 3.2.3. Age and WHO Grade

Based on data from two pediatric series [30,31] with a total of 24 tumors, the Psammomatous (*n* = 6) and Meningothelial (*n* = 5) subtypes were dominant among WHO grade 1 meningiomas in this age group. However, of the 24 tumors, eight were WHO grade 2, constituting a considerably higher proportion than in the general population of patients with spinal meningiomas (33.3% vs. 4.3%). This finding was consistent in both studies, even though patients with neurofibromatosis or multiple meningiomas were excluded in one. There were no WHO grade 3 tumors documented in children [31].

In contrast, two studies [26,27] on the elderly population (>65 and >70) found only three WHO grade 2 meningiomas among 95 (3.2%) tumors. There were no WHO grade 3 tumors. Two other studies [8,34] comparing 70 patients younger than 50, with 127 patients older than 50 years old, could reveal a relative predilection for WHO grade 2 tumors in the younger group (5.7% vs. 0.8%). Similarly, most studies on WHO grade 2 and 3 tumors reported an average patient age significantly lower than that of the general meningioma population. For example, a study of 12 patients with WHO grade 2 Clear cell spinal meningiomas reported an average age of 28.8 years [35], and two other studies with mixed higher grades (WHO grades 2 and 3) in 16 and 25 patients reported a mean age of 52.8 [36] and 46.6 years [37], respectively. In one study, reporting on 13 patients with WHO grade 2 atypical meningiomas, a mean age of 65.7 years was reported [38].

In summary, WHO grade 1 tumors are by far the most predominant, regardless of the age group, while WHO grade 3 meningiomas are extremely rare. A considerable overrepresentation of higher WHO grade meningiomas (WHO grade 2 and 3) was seen among younger patients.

### 3.3. Tumor Biology

#### 3.3.1. Genomics of Spinal Meningiomas

The current understanding of the genomics of meningioma originates from research on cranial tumors, and only a small number of studies concern spinal meningiomas. We found two articles describing chromosomal and genetic alterations associated with spinal meningiomas, analyzing 14 [39] and 16 tumors, respectively [40]. The first study revealed a tendency for spinal meningiomas to originate from a single cell clone, which in most cases harbored a partial or complete loss of chromosome 22, unlike intracranial tumors, which often exhibited a plurality of origin cells [39]. The authors also reported that close to 1500 genes were differentially expressed between spinal and cranial meningiomas. Among them, 35 genes were typically expressed in spinal meningiomas [39]. These genes included the NR4 and Hox gene families involved in transcription. Other genes were involved in intracellular and extracellular signaling pathways [39]. In the second study, specific chromosomal abnormalities in spinal meningiomas were studied [40]. In line with the findings of the former study [39], the most commonly identified chromosomal aberration was partial or complete loss of chromosome 22 [40]. Other aberrations included losses on 1p, 9p and 10q chromosomes, amplifications on 5p or 17q were also seen. The only WHO grade 2 tumor in this sample had the most abnormalities. This tumor had loss of 22q12, 10q25-qter, and 9p23-pter with concomitant amplification of 5p15-pter and 17q23, indicating that such tumors are more complex and may behave differently than their benign counterparts [40].

Expression of the MMP-9 metalloproteinase has been extensively investigated in intracranial meningiomas, as it has been found to correlate with tumor invasiveness. However, only one of the included studies [41] documented the MMP-9 expression in 58 spinal tumors, of which 44 tested positive, with a strong immunoreactivity identified in 19 of the specimens. Unlike in intracranial meningiomas, higher MMP-9 expression was not associated with higher tumor proliferation as measured by Ki-67/MIB-1 labeling and failed to prove clinically significant.

In summary, most of our understanding of genomic alterations is derived from intracranial meningiomas, since only few studies on the genetics of spinal meningiomas have been carried out. However, separate genetic mechanisms and pathways between cranial and spinal meningiomas have been suggested. A few studies on the genetics of spinal meningiomas failed to meet the inclusion criteria as they had analyzed less than ten samples. This reinforces the need for pre-clinical studies to address the basic sciences surrounding spinal meningiomas. Of the excluded studies, two studies of interest had implicated mutations in the SMARCE1 gene as both the potential cause behind cases of familial multiple spinal meningiomas in the absence of NF2 gene mutations [42], and an essential component of the genetic makeup of Clear cell meningiomas [43]. With the advances in genetics, gene therapy has been studied in animal models of both spinal and intracranial meningiomas [44,45,46,47,48]. Thus far however, targeted therapies (ex: anti-VGEF) in the treatment of intracranial meningiomas have not shown definitive results [44,49,50,51].

#### 3.3.2. Receptor Expression

There were seven studies on receptor expression in spinal meningioma cells. All studies analyzed progesterone receptor (PR) expression, four estrogen receptors (ER) and one androgen receptor (AR) expression (Supplementary File S3, Table S5).

Of the 721 tumors included in these seven studies, the PR status was studied in 331, with 58.3% (*n* = 193) showing positivity. Four of the studies presented data on the degree of PR immunoreactivity. The scores of 1, 2, and 3 corresponded to <15%, 15–50%, and >50% of the nuclei staining positive, respectively. Of the 106 tumors analyzed, the majority (*n* = 44, 41.5%) had a score of 3, followed by 2 (*n* = 38, 35.9%), and only a minority (*n* = 24, 22.6%) scored 1 for PR. Hence, most PR-positive tumors tested strongly for PR.

The ER-status was examined in four studies combining 118 tumors. Only nine tumors (7.6%) tested weakly positive with a score of 1, all originating from one study.

One study on androgen receptor (AR) expression in 30 spinal meningiomas found an intermediate expression with a score of 2 in all tumors.

In summary, contrary to the expression of ER, PR-positivity seems to be prevalent among spinal meningiomas. AR was expressed in all tumors from a single study. Further investigation is warranted to validate these findings. Although the expression of sex-hormone receptors in cranial meningiomas has been extensively studied, solid evidence on their value as prognostic markers or tools in targeted therapy is currently lacking.

#### 3.3.3. MIB-Index

During the past few decades, tumor proliferation markers such as Ki-67/MIB-1 have become clinical routine providing prognostic information in oncology. However, relatively few of the studies in this review reported data on tumor proliferation markers. The slow growth of spinal meningiomas is matched by low levels of proliferation markers providing limited information. There were 13 studies reporting data on Ki-67/MIB-1, but only eight of them were representative for the whole meningioma population. Two of the five remaining selectively addressed pediatric cases, where higher WHO grades were overrepresented, and the three others only included higher-grade tumors. The eight studies that were representative compiled a total of 681 tumors. Based on 284 of these (from four different studies), a mean MIB-1 index of 1.9%, with a range from 0.5 to 20% could be calculated. A median MIB-1 index of 4 and 4.5% was stated by two other studies. (Supplementary File S3, Table S5). Moreover, based on the data provided by three studies on 203 tumors [34,41,52], it was noted that 193 (95%) had a labeling index lower than 4%, while only ten (5%) had higher than 4%.

To evaluate the theory that higher-grade spinal meningiomas tend to have higher MIB-1 indices, data from four different studies that presented evidence on Ki-67/MIB-1 in WHO grade 2 and 3 tumors was pooled [6,38,41,53]. A total of 58 tumors (8 grade 3 and 50 grade 2) were identified. Four tumors were excluded as they were confirmed to be spinal metastases of WHO grade 2 intracranial meningiomas. MIB-1 indices ranged from 1 to 20% across the 58 tumors with an average of 9.9%, considerably higher than that of WHO grade 1 (1.9%). Notably, one of the studies contained all 8 WHO grade 3 tumors of this sample and reported the highest individual mean MIB-1 index [6]. A fifth study exclusively limited to higher WHO grades, and specifically to clear cell meningiomas (*n* = 12), revealed that 6 of the tumors had a MIB-1 index over 5% [35]. Despite the overrepresentation of higher-grade tumors in the two studies on 24 pediatric tumors, a surprisingly low overall mean MIB-1 index of 1.6% was calculated with indices ranging from 1 to a maximum of 4%.

Spinal meningiomas are slow growing tumors with low MIB-1 indices. As expected, higher grade tumors had higher MIB-1 indices. Surprisingly, the pediatric population, with more higher-grade tumors (33.3% vs. 4.3%) had an average MIB-1 index lower than that of the whole cohort. An inherent limitation of the analysis of proliferation rates lies in the technical differences in the analysis of MIB-1 indices. As the technical aspects of such measurements are rarely reported, these differences cannot be accounted for.

#### 3.3.4. Neurofibromatosis Type 2 and Other Genetic Conditions

Neurofibromatosis type 2 (NF2) is a known predisposing factor for the development of spinal meningiomas. In this material, 20 studies with 4203 patients documented a total of 66 cases of NF2. Fourteen of the studies, including 1027 patients, were representative of the whole population of patients with spinal meningiomas. These studies reported a total of 30 NF2 cases, representing 2.9% of the study population. Individual age data were available for seven patients yielding a median/mean of 28/27.8 years (range: 18–39). Similarly, a study on four Neurofibromatosis type 2 patients with ten spinal meningiomas revealed a median age of 25 years (22–26) [54].

Three studies only including adults (>18 years) reported 28 cases among 3063 patients (0.9%) [4,55,56]. The lower percentage in these studies may indicate that a significant number of these patients are diagnosed before reaching adulthood. In fact, a study on pediatric cases (<18 years) reported four cases of NF2 among ten patients (40%) [30]. Moreover, two studies explicitly mentioned the increase in the NF2/non-NF2 ratio with a decrease in age [34,41]. In another study, it was noted that all five NF2 patients included in the cohort of 80 patients were below 40 years [8]. Finally, the authors of the study with the highest recorded NF2 prevalence (15.8%) reported that the patients with NF2 were significantly younger [3].

Genetic conditions other than NF2 were rarely reported. Neurofibromatosis type 1 (NF1) was reported in one patient [57], while in another study 14 of 103 patients were classified as having either NF1 or NF2 without specifying the distribution between the two diagnoses [58]. One spinal meningioma patient was diagnosed as having Von Hippel-Lindau syndrome (VHL) [59]. The rarely reported concurrences of spinal meningiomas with phacomatoses other than NF2 seem to be the results of coincidence rather than true associations.

In summary, the prevalence of NF2 in spinal meningioma patients is close to 3%. However, the incidence is higher in younger age groups, reaching up to 40% [30,34]. Spinal meningiomas are uncommon among the younger population but are often associated with an underlying diagnosis of NF2. However, the lack of unified diagnostic criteria and lack of routine genetic testing [36] may affect these numbers. In young patients (<30 years) presenting with spinal meningioma, genetic work-up to rule out NF2 should be considered. The co-occurrence of spinal meningiomas with genetic conditions other than NF2 is rare and likely coincidental.

### 3.4. Tumor Location and Shape

Tumor locations were reported in relation to the adjacent vertebrae. The heterogeneity in reporting the locations of spinal meningiomas does not allow for pooling and analysis of the data. Instead, the data are presented as reported by the authors (Supplementary File S3, Table S6) and limited to estimates of the relative distribution along the spinal canal. Studies with inclusion biases regarding the craniocaudal or axial locations were excluded in the corresponding sections.

#### 3.4.1. Craniocaudal and Axial Tumor Locations

In total, 73 studies reported on the craniocaudal location of 4232 meningiomas. Craniocervical tumors (*n* = 58) were excluded. Most tumors were thoracic (*n* = 2867; 68.7%), followed by cervical (*n* = 984; 23.6%), lumbar (*n* = 164; 3.9%) and sacral (*n* = 6; 0.1%). In studies reporting cervicothoracic and thoracolumbar locations, the former location was twice as common as the latter (100 cases vs. 53), while purely thoracic meningiomas still constituted the majority of cases (*n* = 1154; 66.0%). However, both pediatric series in this review reported a higher proportion of cervical tumors compared to thoracic ones. This resonates with the reported finding [34].

Forty-four studies representatively reported axial locations of 2789 tumors. Lateral tumors were the most common with 1146 reported in this location. However, in studies where ventrolateral and dorsolateral tumors were separately reported, lateral location failed to show superiority to similar extents. This may indicate that part of the predominance may be attributed to the combination of other adjacent axial locations with the lateral subgroup. Albeit ventral and dorsal tumors were close to equally prevalent when pooling all 44 studies, these results were not consistent at the level of individual studies.

#### 3.4.2. Dumbbell Formation

Spinal meningiomas are principally intradural extramedullary tumors. They sometimes extend into the extradural space and take the shape of a dumbbell as a result of being compressed centrally as they pass through the neuroforamen. It is worth noting that the dumbbell shape is much more typical of spinal schwannomas growing along nerve roots. In this review, not all studies reported the incidence of dumbbell tumors. A total of 67 tumors of this type were reported across 26 studies. Considering the total number of tumors examined in these studies (*n* = 1696), the proportion of dumbbell tumors is estimated at 4.3%. However, this number is subject to significant publication and selection biases and may not be representative of the population, as it cannot be known whether the remaining studies simply refrained from publishing this information.

### 3.5. Tumor Presentation

#### 3.5.1. Presenting Symptoms and Symptom Duration

Due to the inconsistency of the terminology used and the overlap between symptoms within and across studies, we chose not to present detailed quantitative data on the prevalence of the different symptoms in patients with spinal meningiomas. Rather, an overview of the most common signs and symptoms, as stated by the authors, is presented. For every study mentioning this information, we extracted the three most common presenting symptoms in order of prevalence. In an attempt to homogenize the data across studies, symptoms were grouped under the following categories: motor dysfunction (including all forms of paresis and paralysis), sensory dysfunction (including paresthesia, hypesthesia or anesthesia), pain (including both local and radicular pain), gait disturbance, and finally bladder or bowel dysfunction.

A total of 50 studies on 6041 patients reported the prevalence of different presenting symptoms (Supplementary File S3, Table S7). Pain, motor, and sensory dysfunction were almost equally cited as the single most common presenting symptom. Altogether, motor dysfunction was included in the top three most common symptoms in 46 out of the 50 studies (92%), followed by sensory symptoms (78%), pain (76%), gait disturbance (42%), and bladder or bowel dysfunction (28%) (Table 2).

There were 29 studies with 2008 patients reporting the average duration of symptoms from onset to diagnosis or treatment. One study instead presented the median value. Adjusting for the number of patients in each of the 29 studies resulted in an overall average symptom duration of almost a year (13.6 months). The ranges for the symptom durations were mentioned in 24 studies and were between 0 and 204 months, reflecting a notable variation within as well as between studies. The delays in diagnosis likely reflect the challenges in diagnosing a disease with an insidious onset and a gradual neurological decline resembling age-related processes. In the two pediatric series in this material [30,31], patients below 18 years of age had a considerably shorter time to diagnosis when compared to the mixed population (8 vs. 13.6 months). One study examined the relation between time to diagnosis and axial location of the tumor but found no association [60]. Another study found a significant relation between mean symptom duration and WHO grade, suggesting that patients with a higher WHO grade had a more obvious development of symptoms [35]. In line with this, a study including only WHO grade 2 spinal meningiomas reported a mean duration of symptoms of 6.5 months, a value significantly lower than the overall average of 13.6 months. In contrast, a study on symptom duration between lower-and higher-grade meningiomas in 336 patients found no significant differences [61]. Schaller et al. compared male and female spinal meningioma patients and found that male patients tended to present later than females [62]. Asymptomatic or incidental meningiomas were reported in 12 studies with 1377 patients and incidences ranged from 2.5 to 30.8%, with 86 tumors detected in total. The pooled proportion across the 12 studies was estimated at 6.3%.

#### 3.5.2. Correlation of Specific Symptoms with Location and Other Factors

Five studies examined the possible relationships between tumor location and symptoms. Regarding axial location and presenting symptoms, two studies found no correlations [63,64]. However, one study found that pain was significantly more common in posterior tumors and motor deficits in anterior ones, sensory deficits were as common in both locations [60]. Regarding craniocaudal location, three studies found motor dysfunction to be significantly more common in thoracic tumors [63,65,66]. Motor deficits were associated with the sacral location in only one study [65]. Yet another study associated sensory deficits to the thoracic location, while they found pain to be more common in cervical cases. In addition, all patients with bladder or bowel dysfunction in the study had thoracic spinal meningiomas, but the results were not significant to make that association [63].

Age was positively correlated to motor symptoms in three studies [4,55,65], to sensory deficits in two studies—one of which with non-significant results [4,65]—gait, and bladder and bowel deficits in one study [55], and to none of the symptoms in another study [63]. In one of these studies including a total of 2844 patients, the authors stated that the symptoms were more severe in the elderly population.

One study only addressed the correlation of sex with specific symptoms but found no statistically significant differences [63]. Another study found that WHO grade 2 and 3 tumors were associated to lower limb weakness to a greater extent than WHO grade 1 tumors [35].

#### 3.5.3. Degree of Tumor Compression and Correlation to Symptoms

Information on spinal cord compression or tumor occupancy of the spinal canal with overall mean, median, or range values were presented in 13 studies [3,4,28,56,58,60,63,65,67,68,69,70,71]. The studies reported spinal cord compression to varying degrees and while the definitions of mild, moderate, or severe spinal cord compression varied between studies, the distribution between the categories were similar.

In addition, two studies analyzed the relation between compression and symptomatology. While one of them found a positive correlation between the degree of spinal cord compression and the occurrence of both motor and gait disturbances separately [69], the other found no correlations [65]. The former study also demonstrated a higher correlation between tumor occupancy, rather than spinal cord compression, and motor, gait, and bladder or bowel deficits [69]. A significant tumor occupancy cutoff value of 65% was proposed to indicate increased prevalence of symptoms [69]. Another study estimated this cutoff value at 63.7% in relation to the occurrence of subjective weakness [63]. In the same study, increased tumor occupancy was associated with sensory dysfunction and bladder or bowel dysfunction, but negatively correlated with pain [63]. A third study looking at the occurrence of paralysis in patients with spinal meningioma could not find a statistically significant correlation to tumor occupancy [70]. Lastly, one study found tumor volume to be positively correlated with the incidence of back pain [65].

In brief, motor dysfunction, sensory dysfunction, and pain are the most common presenting symptoms in patients with spinal meningiomas. Most of these symptoms are more common in elderly patients, patients with thoracic meningiomas, and in patients with greater tumor occupancy of the spinal canal or greater spinal cord compression.

### 3.6. Radiological Diagnosis

#### 3.6.1. General Radiological Features and Findings Described

Twenty studies discussed radiological findings and features associated with spinal meningiomas. On T1-weighted MRI imaging, most of the tumors were isointense, some were hypointense, and very few were hyperintense. Similarly, on T2-weighted MRI, most tumors were isointense. The remainder tended to show hyperintensity rather than hypointensity. Overall, the majority of spinal meningiomas show a strong and homogenous contrast enhancement on MRI. Interestingly, one study found the signal intensity on T2-weighted imaging to be significantly associated with the consistency of the tumor: softer tumors tended to show stronger T2-weighted signal intensities [72].

Seven of these studies also addressed T2-weighted signal changes within the spinal cord in patients with spinal meningiomas. Signal changes were seen in a considerable proportion of patients. Depending on the study, the proportion of patients presenting with signal changes could reach up to 68%, with a median of 43% between all studies. One study reported that most of these changes were of a mild character and only 1% were considered severe [56].

Some authors argued that computed tomography (CT) was useful to assess intratumoral calcification. Otherwise, CT was only used as a substitute for MRI when the latter was contraindicated. One study advocated the use of positron emission tomography (PET) together with a glucose radiotracer to differentiate between spinal meningiomas and schwannomas [73].

#### 3.6.2. Calcification of Spinal Meningioma

Eleven studies reported the proportion of calcified tumors, with numbers ranging from 2.6 to 75%, and the pooled average was estimated to 23.7% (159 of 672 tumors). Calcification was typically diagnosed on CT. Some authors graded the degree of calcification by subgrouping the tumors into severely, moderately, or mildly calcified, while others reported the mean radiodensity in Hounsfield units (HU). Mean radiodensity values were presented in four studies [30,70,72,74] and ranged from 45 in a pediatric study [30] to 628.7 HU in a study primarily targeting adults [70]. Whilst most authors evaluated calcification in HU on CT, some based their evaluation on intraoperative tumor consistence. The terms calcified and ossified are often used interchangeably. Interestingly, two studies [70,75] noticed that intratumoral calcification patterns matched the hypointense areas in tumors that were heterogeneously enhancing on contrast MRI, possibly due to a reduced uptake of contrast in calcified regions. One study [70] mentioned that calcified tumors that were homogenously contrast enhancing tended to exhibit a more homogenous, diffuse, and low-grade calcification pattern. A third study [74] found a positive correlation between the time from onset of symptom to presentation at hospital with the degree of calcification in HU. In this context, it is worth noting that the lowest rates of calcification were reported in a cohort of pediatric patients [30].

#### 3.6.3. Differences and Similarities between Spinal Meningiomas and Schwannomas

There were seven studies investigating the differences between spinal schwannomas and meningiomas [73,76,77,78,79,80,81]. The dural tail sign was typically associated to meningiomas [73,79,80,81] and the dumbbell shape of the tumor to schwannomas [73,77,79]. Two studies pointed out that intratumoral calcifications were significantly more common in spinal meningiomas [73,80] and another two found that cystic degeneration of the tumor was more commonly associated with spinal schwannomas [77,79]. The average size of schwannomas was significantly larger than that of meningiomas in three studies [73,79,80]. However, in a fourth one, no differences could be found [76].

Differences in the location of the tumor were investigated in six studies [73,77,78,79,80,81]. Lumbar tumors were predominantly schwannomas, and thoracic and cervical tumors were mainly meningiomas.

One study investigated differences between the two tumor types on T1-weighted imaging but found no significant correlations [80]. Two studies found that spinal schwannomas showed higher intensities on T2-weighted MRI compared to meningiomas [78,81], while one study demonstrated the opposite [80]. Regarding tumor enhancement on contrast MRI, one study found that spinal schwannomas were associated with both a stronger and more heterogenous enhancement compared to meningiomas [81]. However, another study failed to confirm this finding [80].

As mentioned previously, one study used PET to differentiate between the tumor types and found differences in the maximal Standardized Uptake Values (SUV max). The authors argue the possibility to differentiate between the benign and more aggressive or malignant subtypes [73].

In two studies, the combined use of multiple variables to create a mathematical model resulted in a higher accuracy in differentiating between the two tumor types [79,81].

### 3.7. Non-Surgical Primary, Adjuvant, or Salvage Therapy

#### 3.7.1. Use and Trends Related to Non-Surgical Treatments

Twenty-seven studies reported on the use of non-surgical alternative treatments in 527 spinal meningioma patients. The absolute majority received either conventional fractionated radiotherapy or stereotactic radiosurgery. The uses of these two modalities were reported for 58.4% and 40% of the tumors, respectively. Only four patients received other treatments: one received chemotherapy alone [67], one had combined chemo- and radiotherapy [67], one had combined Receptor Tyrosine Kinase inhibitor and radiotherapy [59], and the last one received brachytherapy [82].

Non-surgical treatment options were used as a primary means in 34.2%, as postoperative adjuvant in 34.9%, and as a salvage therapy in 10.4% of the patients. Whether the therapy was used as primary or adjuvant was unknown in 20.5% of the cases (Supplementary File S3, Table S8).

In 21 of the studies, only a fraction of patients received non-surgical options as part of the treatment plan, with proportions ranging from 0.6% to 23.5%, with a mean of 7.7%. However, six studies included patients having received nonsurgical treatments, specifically stereotactic radiosurgery [82,83,84,85,86,87]. In the largest of these studies, 298 spinal meningiomas were collected, where therapeutic radiation was used as part of the treatment plan. The goal was to determine the trends in the utilization of such treatment modalities over time. The authors found that the use of radiation as the primary treatment remained unchanged at 0.8%, while its use as an adjuvant to surgery gradually increased from 0.5% in 2004 to around 2% almost ten years later [82].

The indications for nonsurgical treatment modalities were present in 22 studies. In 11 studies, patients received radiation therapy or stereotactic surgery on the premises of tumors with higher WHO grades. Primary or secondary recurrence or regrowth of the tumor as well as subtotal resection were stated as the main treatment indication in nine and seven studies, respectively. Other indications for non-surgical treatment, such as increased risk of perioperative complications or postoperative neurological deficit, NF2, and multiple tumors were also reported (Supplementary File S3, Table S8).

#### 3.7.2. Benefits and Risks Associated with Radiotherapy

The outcomes of alternative therapies, either as primary or adjuvant treatment, were reported in 16 studies (Supplementary File S3, Table S8). Five studies reported outcomes of stereotactic radiosurgery in patients with spinal meningioma, with a follow-up time of around 30 months [83,84,85,86,87]. Among the 101 spinal meningiomas treated with this modality, local control was achieved in the majority, with only five tumors experiencing recurrence (5%). Four of the five recurrences occurred in the same patient, who had a spinal meningioma attributed to previous radiation exposure [85]. In most of the patients, the neurological status either stabilized or improved after treatment. The reported complications attributed to radiosurgery were nausea [84], radiation-induced cord toxicity [83,86], and transient neurological deficits [84].

The two studies with the largest patient cohorts in this section only presented data on survival rather than recurrence or evolution of symptoms [32,82]. The two studies compared patients who had received radiation as part of their treatment with those who had not, in terms of overall survival, and found similar compelling results. Patients receiving radiation had a statistically significant decreased survival compared to those who did not receive radiation [32]. The results were identical even when only higher WHO grades were considered [82]. However, none of the studies adjusted for other plausible confounders, such as age, recurrence state, underlying NF2 diagnosis, etc.

Recurrence/regrowth was present in eight different studies on 26 tumors treated with conventional radiotherapy. Treatment failure was observed in nine of the tumors (34.6%). In one of the studies, authors compared patients who had received adjuvant radiotherapy with those who had not and found a significantly reduced recurrence rate in the formers [36]. In another study, two patients who had received radiotherapy as part of their treatment plan experienced severe complications, with one case of neurological deficits prompting treatment arrest and a case of arachnoiditis that was diagnosed six months after radiotherapy [53]. Information on the recurrence status of these two tumors was not present. Other complications related to radiotherapy include radiation necrosis, panic attacks, and constipation [36].

As the mainstay of spinal meningioma treatment is radical surgery, there are limited indications for adjuvant radiation, let alone other alternative therapies. Patients who may benefit from such treatment modalities are, hence, typically those in whom surgery is contraindicated or those with sub-totally resected tumors, higher WHO grade (2 or 3) tumors, or underlying neurocutaneous disorders. Nonetheless, not all authors offer adjuvant therapy in patients that fit these indications. Two authors avoided the use of adjuvant therapy even for patients with WHO grade 2 meningiomas [35,38]. Although one study reported no tumor recurrence [38], the other reported recurrence in 42% of patients [35]. Contradictory outcomes were also reported in studies offering adjuvant therapy. While some reported no complications or recurrence of tumors [36,62,88], others encountered major adverse events and did not benefit from the treatment [37,53,59,89,90]. Hence, no clear conclusions can be drawn regarding the use of adjuvant radiation for the targeted patient groups.

Although the results of radiosurgery for the treatment of spinal meningiomas are promising, with tumor control achieved in the majority of patients, most studies are limited by short follow-up times and small patient cohorts [83,84,85,86,87]. Inversely, the studies that cover the topic of alternative therapies may introduce Berkson’s bias, which may underestimate the treatment effect, as only a targeted population with often higher comorbidity rates is offered such treatment modalities. This is highlighted by the various indications extracted, such as NF2, lower extent of resection, or higher WHO grade, all of which may, per se, result in worse outcomes. Similarly, two studies found radiation to be significantly associated with shorter survival in different patient groups [32,82]. Supposing these results derive from an association rather than causation, this rationale would validate the fact that patients receiving radiation are intrinsically sicker, not because they were treated with radiation but because of other confounders at baseline. This prevents generalization of the results and limits comparability with the conventional surgical treatment, an issue that may be resolved with appropriate study designs and thorough stratification. Similarly, chemotherapy, a treatment option typically reserved for high-grade tumors, was only reported as a treating method in three of the patients included in this review. While research in this area has made progress with regard to intracranial meningiomas, little data exist to support the use of chemotherapy in the treatment of spinal meningiomas. Currently, the evidence is limited to sporadic case reports [91,92]. Until more thorough studies with long-term evaluation of these alternative treatment modalities are presented, we argue that spinal meningiomas will remain a “surgical disease”.

Finally, when the appropriate indications align, and the use of alternative therapies is deemed necessary, a scrupulous risk and benefit assessment should consider possible complications. Reported complications associated with radiation exposure of the spinal cord include cord toxicity and neurological deficits [53,83,84,86]. Radiation-induced myelopathy is one of the most frequently reported complications, occurring in 0.2% to 5% of cases [93]. Other non-specific complications include nausea, radiation necrosis, constipation, and panic attacks [36].

## 4. Conclusions

Spinal meningiomas are associated with a clear female preponderance (1:2.7) and a peak incidence around the seventh decade of life. An interregional variability regarding both sex and age distributions is present. WHO grade 1 tumors are by far the most dominant, while WHO grade 2 and 3 spinal meningiomas are scarce. The most common histological subtype of meningioma across studies were, in order, the Psammomatous, Meningothelial, Transitional, and Fibrous subtypes. A significant overrepresentation of both male patients (62.0% vs. 27.17%) and higher WHO grade tumors (33.3% vs. 4.3%) were found among younger cohorts.

Overall, spinal meningiomas are slow growing tumors. This feature is confirmed by the low MIB-1 indices, around 1.9% on average. Despite the higher proportion of higher-grade tumors among the pediatric population, on average, the MIB-1 indices in this cohort were slightly lower (1.6% vs. 1.9%).

Patients with spinal meningiomas most commonly presented with motor, sensory symptoms, and pain. The evidence indicates that most of these symptoms are more common in elderly patients, patients with thoracic meningiomas, and those with greater tumor occupancy of the spinal canal or greater spinal cord compression.

The mainstay of spinal meningioma treatment is radical surgery. Hence, there are limited indications for non-surgical treatment options. Although some studies have reported promising results, until more solid findings with long-term evaluation of such treatment modalities are presented, spinal meningiomas will remain a “surgical disease”.

## Figures and Tables

**Table 1:** Inclusion and exclusion criteria applied during the article selection process.

Criteria	Inclusion	Exclusion
Study type	Observational/experimental, peer-reviewed, human studies	Case report, review, editorial, letters, and conference abstracts
Publication date	From 1 January 2000 to 1 January 2022	n/a
Language	English	All other languages
Population	All sorts of cohorts with at least ten patients	Studies with less than ten patients
Intervention	All interventions for both treatment and diagnosis of the tumor	n/a
Comparator	n/a	n/a
Outcome	Any outcome clearly reported	n/a

**Table 2:** Table showing the distribution of the three most common symptoms among the 50 studies where at least the most common symptom was presented.

	Most Common Symptom (*n* = Number of Studies)	2nd Most Common Symptom (*n*)	3rd Most Common Symptom (*n*)	Times Mentioned in the Top 3 (% among Studies)
Motor dysfunction	19	16	11	46 (92%)
Sensory dysfunction	17	16	6	39 (78%)
Pain	18	10	10	38 (76%)
Gait disturbance	2	11	8	21 (42%)
Bladder and/or bowel dysfunction	1	2	11	14 (28%)
Data not available	0	2	5	-

## Supplementary Materials

The following supporting information can be downloaded at: https://www.mdpi.com/article/10.3390/cancers14246251/s1, File S1: PRISMA checklist (part 1); File S2: Search strategy; File S3: Figures and tables: Figure S1: Association between the recurrence rate and the follow-up duration, with the associated Pearson correlation coefficient R and p-value; Figure S2. Funnel plot showing the distribution of studies comparing the recurrence rate among low vs. high WHO grade spinal meningiomas; Figure S3. Funnel plot showing the distribution of studies comparing the recurrence rate among ventral vs. non-ventral spinal meningiomas; Figure S4. Funnel plot showing the distribution of studies comparing the recurrence rate spinal meningiomas operated with Simpson grade 1 vs. grade 2 resection; Figure S5. Funnel plot showing the distribution of studies comparing the recurrence rate spinal meningiomas operated with Simpson grade 1 and 2 vs. grade 3, 4, and 5 resections; Table S1: Baseline characteristics and study inclusion under each of the sections of this review; Table S2. Risk of bias assessment; Table S3. Epidemiology; Table S4. Histopathology; Table S5. Genetics and immunohistochemistry; Table S6. Tumor location; Table S7. Presenting symptoms; Table S8. Non-surgical treatment options; Table S9. Surgical treatment of spinal meningniomas; Table S10. Intraoperative neuromonitoring; Table S11. Perioperative complications; Table S12. Neurological outcomes; Table S13. Markers of neurologic outcomes as described by the included studies; Table S14. Recurrence rate and markers of recurrence.
