# Supplementary material for: Current Knowledge on Spinal Meningiomas Epidemiology, Tumor Characteristics and Non-Surgical Treatment Options: A Systematic Review and Pooled Analysis (Part 1)
Source: Cancers (Basel). 2022 Dec 19;14(24):6251. doi: 10.3390/cancers14246251 (PMC9776907; doi:10.3390/cancers14246251)
Supplement: Supplementary file 1 [file cancers-14-06251-s001.zip › Supplementary file S3, Supplementary figures and tables.pdf]

Supplementary File S3: figures and raw data tables

Table S1. Baseline characteristics and study inclusion under each of the sections of this review.

Title	Study ID	Location	Overall, ROB score (6p)	Qualitative ROB	More information on the cohort	Epidemiology	Race	Histopathology	Genetics	Receptors	Tumor location	Presenting symptoms	Radiology	Surgical treatment	Intraoperative monitoring	Operative complications	Alternative treatment	Outcome and HRQOL	Recurrence
Functional Outcome After Spinal Meningioma Surgery. A Nationwide Population-Based Study	Champeaux-Depond 2022	France	5	LOW	Only adults (>18 years)	1	0	0	0	0	0	1	0	0	0	0	0	1	0
The impact of body mass index and height on risk for primary tumours of the spinal cord, spinal meninges, spinal and peripheral nerves in 1.7 million norwegian women and men: a prospective cohort study.	Gheorghiu 2022	Norway	5	LOW	Only patients aged between 18 and 80 years	1	0	0	0	0	0	0	0	0	0	0	0	0	0
Clinical characteristics and long-term outcomes for patients who undergo cytoreductive surgery for thoracic meningiomas: a retrospective analysis.	Ampie 2021	United States	5	LOW	Only thoracic spinal meningiomas were included	1	0	1	0	0	1	1	1	1	1	1	1	1	1
Correlations Among Consistency, Computed Tomography Values, and Histopathological Subtypes of Spinal Meningioma	Aoyama 2021	Japan	6	LOW		1	0	1	0	0	1	0	1	1	0	0	0	0	1
Spinal cord compression in relation to clinical symptoms in patients with spinal meningiomas	Corell 2021	Sweden	5	LOW	Only adults (>18 years) were included	1	0	1	0	0	1	1	0	1	0	1	0	1	1
Correlations between preoperative clinical factors and treatment outcome of spinal meningiomas - A retrospective study of a series of 31 cases	Davarski 2021	Bulgaria	6	LOW		1	0	1	0	0	1	1	0	1	1	1	0	1	1

Clinical features and prognostic factors in spinal meningioma surgery from a multicenter study.	Kobayashi 2021	Japan	6	LOW		1	0	1	0	0	1	1	1	1	1	1	1	1	1
Clinical characteristics and management differences for grade II and III spinal meningiomas	Krauss 2021	United States	5	LOW	Only WHO II & III (n=17)<br>However some data on WHO I is presented	1	0	1	0	0	0	0	0	1	0	1	1	1	1
Long-Term Follow-Up and Predictors of Functional Outcome after Surgery for Spinal Meningiomas: A Population-Based Cohort Study	Pettersson-Segerlind 2021	Sweden	6	LOW	Only adults (>18 years) were included	1	0	1	0	0	1	1	0	1	1	1	1	1	1
Long-term outcomes of spinal meningioma resection with outer layer of dura preservation technique.	Saiwai 2021	Japan	5	LOW	Only WHO I and follow-up of 5 years at least	1	0	1	0	0	1	0	0	1	0	1	0	0	1
Surgical results of the resection of spinal meningioma with the inner layer of dura more than 10 years after surgery	Tominaga 2021	Japan	6	LOW		1	0	1	0	0	1	0	0	1	0	0	0	1	1
Age at Diagnosis and Baseline Myelomalacia Sign Predict Functional Outcome After Spinal Meningioma Surgery	Wach 2021	Germany	6	LOW		1	0	1	0	0	1	1	1	1	1	1	0	1	1
Spinal Meningiomas: Influence of Cord Compression and Radiological Features on Preoperative Functional Status and Outcome	Baro 2021	Italy	5	LOW	Only patients aged between 18 and 85 years were included	1	0	1	0	0	1	0	1	1	1	1	0	1	1
Epidemiological features of meningiomas: a single Brazilian center's experience with 993 cases.	Colli 2021	Brazil	6	LOW		1	0	1	0	0	0	0	0	0	0	0	0	0	0
Functional outcome and morbidity after microsurgical resection of spinal meningiomas.	Kilinc 2021	Germany	6	LOW		1	0	1	0	0	1	1	0	1	1	1	0	1	1

Characteristics of cases with and without calcification in spinal meningiomas.	Kobayashi 2021	Japan	6	LOW		1	0	1	0	0	1	0	1	0	0	0	0	1	0
Improvement in Quality of Life Following Surgical Resection of Benign Intradural Extramedullary Tumors: A Prospective Evaluation of Patient-Reported Outcomes.	Newman 2021	United States	6	LOW		0	0	0	0	0	0	0	0	0	0	0	0	1	0
Assessing the Utility of 18F-Fluorodeoxyglucose Positron Emission Tomography in the Differential Diagnosis Between Spinal Schwannomas and Meningiomas	Ono H. 2021	Japan	6	LOW		1	0	0	0	0	1	0	1	0	0	0	0	0	0
Predictive Value of Heterogeneously Enhanced Magnetic Resonance Imaging Findings With Computed Tomography Evidence of Calcification for Severe Motor Deficits in Spinal Meningioma	Ono K. 2021	Japan	6	LOW		1	0	1	0	0	1	1	1	1	0	0	0	1	0
Health-related quality of life and return to work after surgery for spinal meningioma: A population-based cohort study	Petersson-Segerlind 2021	Sweden	5	LOW		0	0	0	0	0	0	0	0	0	0	0	0	1	0
Epidemiology and survival of patients with spinal meningiomas: A SEER analysis	Cao 2021	China (US population though)	6	LOW		1	1	1	0	0	0	0	0	0	0	0	1	0	0
Clinical features and surgical outcomes of high-grade spinal meningiomas: Report of 19 cases and literature review.	Han 2020	China	5	LOW	Focus on high grade spinal meningiomas (n=20), however some data on lower grade spinal meningiomas is	1	0	1	0	0	1	1	0	1	1	1	1	1	1

					mentioned to some extent.														
Functional outcome after surgical treatment of spinal meningioma.	Hohenberger 2020	Germany	6	LOW		1	0	1	0	0	1	1	0	1	1	1	0	1	1
Spinal meningiomas: is Simpson grade II resection radical enough?	Voldřich 2020	Czech Republic	5	LOW	Tumors treated by Simpson grade 3 excision (n=8) were excluded. Simpson grade 1 was not used.	1	0	0	0	0	1	1	0	1	1	1	0	1	1
Spinal meningiomas: Treatment outcome and long-term follow-up.	Kwee 2020	Netherlands	5	LOW		1	0	1	0	0	1	1	0	1	1	1	1	1	1
Differentiating between spinal schwannomas and meningiomas using MRI: A focus on cystic change.	Lee 2020	South Korea	6	LOW		1	0	0	0	0	1	0	1	0	0	0	0	0	0
Low recurrence after Simpson grade II resection of spinal benign meningiomas in a single-institute 10-year retrospective study.	Naito 2020	Japan	5	LOW	Only WHO I with a follow up of 2 years at least	1	0	0	0	0	1	0	0	1	0	0	0	1	1
Comparative clinical and genomic analysis of neurofibromatosis type 2-associated cranial and spinal meningiomas.	Pemov 2020	United States	5	LOW	Only NF2 patients were included	1	0	0	0	0	0	0	0	0	0	0	0	0	0
New Insights into Expression of Hormonal Receptors by Meningiomas.	Portet 2020	France	6	LOW		1	0	1	0	1	0	0	0	0	0	0	0	0	0
Estrogen and progesterone receptor in meningiomas: An immunohistochemical analysis.	Telugu 2020	India	6	LOW		0	0	0	1	1	0	0	0	0	0	0	0	0	0
Differences and characteristics of symptoms by tumor location, size, and degree of spinal cord compression: a retrospective	Yamaguchi 2020	Japan	5	LOW		1	0	1	0	0	1	1	0	0	0	0	0	0	0

study on 53 surgically treated, symptomatic spinal meningiomas																			
Recurrence of spinal meningiomas: analysis of the risk factors.	Maiuri 2020	Italy	5	LOW	Mixed spinal and intracranial meningiomas. Only Simpson I & II and adults (>20 years)	0	0	0	0	0	0	0	0	0	0	0	0	0	1
Treatment outcomes of 17 patients with atypical spinal meningioma, including 4 with metastases: a retrospective observational study.	Noh 2019	South Korea	5	LOW	Only atypical meningiomas (WHO II)	1	0	1	0	0	1	1	0	1	1	0	0	1	1
Microscopic keyhole technique for surgical resection of thoracic spinal meningioma via an oblique placing tube	Xu 2019	China	4	MODERATE	Only thoracic with a follow-up of 6 months at least	1	0	1	0	0	1	0	0	1	1	1	0	1	1
Magnetic resonance imaging spectrum of spinal meningioma.	Yeo 2019	South Korea	6	LOW		1	0	1	0	0	1	1	1	0	0	0	0	0	1
The outcome of patients with surgically treated meningioma in England: 1999-2013. A cancer registry data analysis.	Brodbelt 2019	United Kingdom	5	LOW	Mixed intracranial/spinal	0	0	0	0	0	0	0	0	0	0	0	0	1	0
Long-Term Update of Stereotactic Radiosurgery for Benign Spinal Tumors.	Chin 2019	United States	5	LOW	Only patients with radiosurgery	0	0	0	0	0	1	1	0	0	0	0	1	0	0
Surgical outcome of spinal meningioma, single institute experience	Elkatatny 2019	Egypt	4	MODERATE		1	0	1	0	0	1	1	0	1	1	1	0	1	0
Spinal Clear Cell Meningioma: Clinical Study with Long-Term Follow-Up in 12 Patients.	Li 2019	China	5	LOW	Only clear cell meningiomas (WHO II), however some data regarding WHO I tumors is present	1	0	1	0	1	0	0	1	1	0	1	0	0	0
WHO grade, proliferation index, and progesterone receptor expression are different according to the location of meningioma.	Maiuri 2019	Italy	6	LOW		0	0	1	0	1	0	0	0	0	0	0	0	0	0

Survival in Patients with High-Grade Spinal Meningioma: An Analysis of the National Cancer Database.	Wright 2019	United States	5	LOW	Only WHO II & III and adult (>18 patients)	1	1	1	0	0	0	0	0	0	0	0	0	0	0
Trends in the utilization of radiotherapy for spinal meningiomas: insights from the 2004-2015 National Cancer Database.	Yolcu 2019	United States	5	LOW	Only patients with radiosurgery or radiotherapy	0	0	0	0	0	0	0	0	0	0	0	1	0	0
Spinal Meningiomas Prognostic Evaluation Score (SPES): predicting the neurological outcomes in spinal meningioma surgery.	Frati 2019	Italy	6	LOW		1	0	0	0	0	0	0	0	0	0	0	0	0	0
Clinical and prognostic features of spinal meningioma: a thorough analysis from a single neurosurgical center.	Hua 2018	China	6	LOW		1	0	1	0	1	1	1	0	1	1	1	0	1	1
Spinal meningiomas - Risks and potential of an increasing age at the time of surgery.	Schwake 2018	Germany	5	LOW		1	0	1	0	0	1	1	1	1	1	1	1	1	1
Spinal meningioma and factors predictive of post-operative deterioration.	Gilard 2018	France	5	LOW	Only patients >18 years were included	1	0	1	0	0	1	1	0	1	0	1	0	1	1
Preliminary algorithm for differential diagnosis between spinal meningioma and schwannoma using plain magnetic resonance imaging.	Iwata 2018	Japan	6	LOW		1	0	0	0	0	0	0	1	0	0	0	0	0	0
Surgical management of spinal meningiomas: focus on unilateral posterior approach and anterior localization.	Onken 2018	Germany	4	MODERATE	Laterally located tumors excluded	1	0	1	0	0	0	0	0	1	0	1	0	1	1
Benefits of spinal meningioma resection	Santos 2018	Brazil	6	LOW		1	0	1	0	0	1	1	0	1	0	1	0	1	0
Differentiating spinal intradural-extramedullary schwannoma	Takashima 2018	Japan	6	LOW		1	0	0	0	0	1	0	1	0	0	0	0	0	0

from meningioma using MRI T(2) weighted images.																			
Surgical management of ventrally located spinal meningiomas via posterior approach.	Notani 2017	Japan	5	LOW	Only ventral tumors	1	0	0	0	0	0	0	0	1	1	1	0	1	1
Clinical features and long-term outcomes of pediatric spinal meningiomas.	Wu 2017	China	5	LOW	Only pediatric <18 years included and all NF2 patients were excluded.	1	0	1	0	0	1	1	0	1	1	1	1	1	1
Proposal of a new radiological classification system for spinal meningiomas as a descriptive tool and surgical guide.	Bayoumi 2017	Turkey and United States	6	LOW		0	0	0	0	0	0	0	1	1	0	0	0	0	0
Spinal meningioma: relationship between degree of cord compression and outcome.	Davies 2017	United Kingdom	6	LOW		1	0	1	0	0	1	1	0	0	0	0	0	1	0
Spinal intradural extramedullary tumors: the value of intraoperative neurophysiologic monitoring on surgical outcome.	Harel 2017	Israel	4	MODERATE	Mixed benign spinal tumors	0	0	0	0	0	0	0	0	1	1	0	0	0	0
Factors Leading to a Poor Functional Outcome in Spinal Meningioma Surgery: Remarks on 173 Cases.	Raco 2017	Italy	6	LOW		0	0	1	0	0	0	1	0	1	0	1	0	1	1
Long-term recurrence rates after the removal of spinal meningiomas in relation to Simpson grades.	Kim 2016	South Korea	5	LOW	Only Simpson Grade 2	1	0	1	0	0	1	0	0	1	0	1	0	1	1
Spinal meningiomas: clinicoradiological factors predicting recurrence and functional outcome.	Maiti 2016	United States	6	LOW		1	1	1	0	0	1	0	1	1	1	1	0	1	1
Unilateral Laminectomy Approach for the Removal of Spinal Meningiomas and Schwannomas: Impact on Pain,	Pompili 2016	Italy	5	LOW	Mixed intradural extramedullary tumors and only hemi-	1	0	0	0	0	1	1	0	1	1	0	0	1	1

Spinal Stability, and Neurologic Results.					laminectomy approach													
Hemilaminectomy for spinal meningioma: A case series of 20 patients with a focus on ventral- and ventrolateral lesions.	Tola 2016	Italy	5	LOW	All patients were operated through a unilateral approach	1	0	1	0	0	1	1	0	1	1	1	0	1	1
Clinical features and prognostic factors of WHO II and III adult spinal meningiomas: analysis of 25 cases in a single center.	Ye 2016	China	5	LOW	Only WHO II & III (some data on WHO I is however mentioned). Only patients >18 years were included.	1	0	1	0	0	1	1	0	1	0	1	1	1	1
Spinal Cervical Meningiomas: The Challenge Posed by Ventral Location.	Lonjon 2016	United Kingdom	5	LOW	Only cervical	1	0	1	0	0	1	1	0	1	1	1	1	1	1
Improved patient quality of life following intradural extramedullary spinal tumor resection.	Viereck 2016	United States	4	MODERATE	Mixed WHO 1 intradural extramedullary spinal tumors	0	0	0	0	0	0	0	0	0	0	0	0	1	0
Does Histologic Subtype Influence the Post-Operative Outcome in Spinal Meningioma?	Zham 2016	Iran	5	LOW		1	0	1	0	0	0	0	0	1	0	0	0	1	0
Surgical management of ventrally based lower cervical (subaxial) meningiomas through the lateral approach: Report on 16 cases.	Aboul-Enein 2015	Egypt	5	LOW	Only cervical ventral	1	0	0	0	0	0	0	0	1	1	1	1	0	1
Simpson Grade I-III Resection of Spinal Atypical (World Health Organization Grade II) Meningiomas is Associated With Symptom Resolution and Low Recurrence.	Sun 2015	United States	5	LOW	Only WHO grade II atypical spinal meningiomas	1	0	1	0	0	0	0	0	1	1	1	1	1	1
Stereotactic radiotherapy for spinal meningiomas and neurinomas.	Golanov 2015	Russia	4	MODERATE	Only patients with radiosurgery	1	0	0	0	0	1	0	0	0	0	0	1	1	0

Outcome of surgical management i spinal meningioma: a study of 48 cases	Haq 2015	Pakistan	4	MODERATE	Patients with severe comorbidities were excluded	1	0	0	0	0	1	1	0	1	1	1	0	1	1
Assessment of the treatment response of spinal meningiomas after radiosurgery focusing on serial MRI findings.	Lee 2015	South Korea	5	LOW	Only patients with radiosurgery	1	0	0	0	0	1	0	1	0	0	0	1	0	0
Hemilaminectomy approach for intradural extramedullary spinal tumors: an analysis of 164 patients.	Turel 2015	India	4	MODERATE	Mixed intracranial/spinal, and only those with a hemilaminectomy performed	0	0	0	0	0	0	0	0	1	1	0	0	0	0
Complications and outcomes of surgery for spinal meningioma: a Nationwide Inpatient Sample analysis from 2003 to 2010.	Ambekar 2014	United States	6	LOW		1	1	0	0	0	0	0	0	0	0	1	0	0	0
Is Simpson grade I removal necessary in all cases of spinal meningioma? Assessment of postoperative recurrence during long-term follow-up.	Tsuda 2014	Japan	5	LOW		1	0	1	0	0	1	1	0	1	1	1	1	1	1
Surgical management of spinal meningiomas: A retrospective case analysis based on preoperative surgical grade	Arima 2014	Japan	5	LOW	Lumbar tumors were excluded	1	0	0	0	0	1	0	1	1	0	0	0	1	1
MRI diagnosis of intradural extramedullary tumors.	Gu 2014	China	6	LOW		1	0	0	0	0	1	0	1	0	0	0	0	0	0
Spinal meningiomas: surgical outcome and literature review.	Riad 2013	France	6	LOW		1	0	1	0	0	1	1	0	1	0	1	0	1	1
Clinicopathological characteristics, hormone receptor status and matrix metallo-proteinase-9 (MMP-9) immunohistochemical expression in spinal meningiomas	Barresi 2012	Italy	6	LOW		1	0	1	0	1	1	0	0	1	0	0	0	0	1
Long-term surgical outcomes of spinal meningiomas.	Nakamura 2012	Japan	5	LOW		1	0	1	0	0	1	0	0	1	0	0	0	0	1

Spinal meningioma in childhood: clinical features and treatment.	Wang 2012	China	5	LOW	Only pediatric <18 included	1	0	1	0	1	1	1	1	1	0	0	1	0	1
Minimally invasive surgery for benign intradural extramedullary spinal meningiomas: experience of a single institution in a cohort of elderly patients and review of the literature.	Iacoangeli 2012	Italy	5	LOW	Only elderly (>68)	1	0	1	0	0	1	1	0	1	1	1	0	1	0
Radiosurgery of spinal meningiomas and schwannomas.	Kufeld 2012	Germany	4	MODERATE	Only patients with radiosurgery	0	0	0	0	0	0	0	0	0	0	0	1	1	0
Spinal meningiomas: age-related features.	Maiuri 2011	Italy	6	LOW		1	0	1	0	1	1	0	0	1	0	0	0	0	1
Spinal meningiomas: recurrence in ventrally located individuals on long-term follow-up; a review of 46 operated cases.	Postalci 2011	Turkey	6	LOW		1	0	1	0	0	1	1	0	1	0	1	0	1	1
Posterior approach to ventrally located spinal meningiomas.	Voulgaris 2010	Greece	5	LOW	Only ventral tumors	1	0	1	1	0	1	0	0	1	1	0	0	0	1
Clinical presentation, histology, and treatment in 430 patients with primary tumors of the spinal cord, spinal meninges, or cauda equina.	Engelhard 2010	United States	5	LOW	Mixed primary spinal cord tumors	1	0	0	0	0	0	1	0	0	0	0	1	0	0
Spinal meningioma surgery in elderly patients with paraplegia or severe paraparesis: a multicenter study.	Sacko 2009	France	5	LOW	Only elderly (>70) with paralysis or severe paresis (grade 3 or 4 ASA)	1	0	0	0	0	1	1	0	1	0	1	0	0	1
Radiological findings of spinal schwannomas and meningiomas: focus on discrimination of two disease entities.	Liu 2009	South Korea	6	LOW		1	0	0	0	0	1	0	1	0	0	0	0	0	0
A less invasive surgical concept for the resection of spinal meningiomas.	Boström 2008	Germany	6	LOW		1	0	1	0	0	1	1	0	1	1	1	0	1	1

Spinal meningiomas: Clinical and therapeutic tonsiderations	Schröder 2008	Germany	6	LOW		1	0	1	0	0	1	1	0	1	0	1	1	1	1
Radiosurgery for benign intradural spinal tumors.	Gerszten 2008	United States	4	MODERATE	Only patients with radiosurgery	1	0	0	0	0	1	0	0	0	0	0	1	0	0
Spinal meningiomas: critical review of 131 surgically treated patients.	Sandalcioglu 2008	Germany	6	LOW		1	0	1	0	0	1	1	0	1	1	1	0	1	1
Management of spinal meningiomas: surgical results and a review of the literature.	Setzer 2007	Germany and United States	6	LOW		1	0	1	0	0	1	0	0	1	0	1	1	1	1
Surgical outcome of spinal canal meningiomas	Yoon 2007	South Korea	5	LOW		1	0	1	0	0	1	0	0	1	0	1	1	1	1
Meningothelioma as the predominant histological subtype of midline skull base and spinal meningioma.	Lee 2006	United States	6	LOW		0	0	1	0	0	0	0	0	0	0	0	0	0	0
Microarray-based analysis of spinal versus intracranial meningiomas: different clinical, biological, and genetic characteristics associated with distinct patterns of gene expression.	Sayagués 2006	Spain	6	LOW		1	0	1	1	0	0	0	0	0	0	0	0	0	0
Results of spinal meningioma surgery in patients with severe preoperative neurological deficits.	Haegelen 2005	France	5	LOW	Only patients with severe presenting symptoms (Grade 3 or 4 Levy Score) were included	1	0	0	0	0	1	0	0	1	0	1	0	1	1
Spinal meningiomas: Evaluation of 41 patients	Peker 2005	Turkey	5	LOW		1	0	1	0	0	1	1	1	1	0	0	0	1	1
Spinal meningioma: relationship between histological subtypes and surgical outcome?	Schaller 2005	Switzerland	6	LOW		1	0	1	0	0	1	1	0	1	0	0	1	1	1

MR imaging features of spinal schwannomas and meningiomas.	De Verdelhan 2005	France	6	LOW		1	0	0	0	0	1	0	1	0	0	0	0	0	0
Results in the operative treatment of elderly patients with spinal meningiomas.	Morandi 2004	France	5	LOW	Only elderly (>70)	1	0	1	0	0	1	0	0	1	0	1	0	1	1
Detection of chromosomal imbalances in spinal meningiomas by comparative genomic hybridization.	Arslantas 2003	Turkey	5	LOW	Only adults (>18)	1	0	1	1	0	1	0	0	0	0	0	0	0	1
Approaches and surgical results in the treatment of ventral thoracic meningiomas. Review of our experience with a postero-lateral combined transpedicular-transarticular approach.	Gambardella 2003	Italy	5	LOW	Only thoracic ventral	1	0	0	0	0	0	1	0	1	0	1	0	1	1
Spinal meningiomas in patients younger than 50 years of age: a 21-year experience.	Cohen-Gadol 2003	United States	6	LOW		1	0	1	0	0	1	1	0	0	0	1	0	1	1
Recurrence of benign spinal neoplasms.	Schick 2001	Germany	5	LOW	Mixed benign spinal tumors	1	0	0	0	0	0	0	0	1	0	0	0	0	1
Review of 36 cases of spinal cord meningioma.	Gezen 2000	Turkey	6	LOW		1	0	1	0	0	1	1	0	1	0	1	1	1	1

Each study was included in at least one of the sections in this review (1 = study included, 0 = study not included), ROB = Risk of Bias, HRQOL = Health-related quality of life, WHO = World Health Organization, NF2 = Neurofibromatosis 2

Table S2. Risk of bias assessment

Study ID	Representativeness of intervention group (1p)	Ascertainment of exposure (1p)	Demonstration that outcome of interest was not present at start of study (1p)	Assessment of outcome (1p)	Was follow-up long enough for outcomes to occur (1p)	Adequacy of follow up of cohorts (1p)	Overall ROB score (6p)	Qualitative ROB	OCEBM	Individual quality Score (IQS)
Gheorghiu 2022	0	1	1	1	1	1	5	LOW	I	1
Champeaux-Depond 2022	0	1	1	1	1	1	5	LOW	II	1
Newman 2021	1	1	1	1	1	1	6	LOW	I	1
Aoyama 2021	1	1	1	1	1	1	6	LOW	III	2
Krauss 2021	0	1	1	1	1	1	5	LOW	III	2
Ampie 2021	0	1	1	1	1	1	5	LOW	II	1
Baro 2021	0	1	1	1	1	1	5	LOW	II	1
Cao 2021	1	1	1	1	1	1	6	LOW	II	1
Colli 2021	1	1	1	1	1	1	6	LOW	II	1
Corell 2021	0	1	1	1	1	1	5	LOW	II	1
Davarski 2021	1	1	1	1	1	1	6	LOW	II	1
Kilinc 2021	1	1	1	1	1	1	6	LOW	II	1
Kobayashi 2021	1	1	1	1	1	1	6	LOW	II	1

Kobayashi 2021	1	1	1	1	1	1	6	LOW	II	1
Ono H. 2021	1	1	1	1	1	1	6	LOW	II	1
Ono K. 2021	1	1	1	1	1	1	6	LOW	II	1
Pettersson-Segerlind 2021	1	1	1	1	1	1	6	LOW	II	1
Pettersson-Segerlind 2021	0	1	1	1	1	1	5	LOW	II	1
Saiwai 2021	0	1	1	1	1	1	5	LOW	II	1
Tominaga 2021	1	1	1	1	1	1	6	LOW	II	1
Wach 2021	1	1	1	1	1	1	6	LOW	II	1
Han 2020	0	1	1	1	1	1	5	LOW	II	1
Hohenberger 2020	1	1	1	1	1	1	6	LOW	II	1
Kwee 2020	1	1	1	1	0	1	5	LOW	II	1
Lee 2020	1	1	1	1	1	1	6	LOW	II	1
Maiuri 2020	0	1	1	1	1	1	5	LOW	II	1
Naito 2020	0	1	1	1	1	1	5	LOW	II	1
Pemov 2020	0	1	1	1	1	1	5	LOW	II	1
Portet 2020	1	1	1	1	1	1	6	LOW	II	1
Telugu 2020	1	1	1	1	1	1	6	LOW	II	1
Voldřich 2020	0	1	1	1	1	1	5	LOW	II	1

Yamaguchi 2020	1	1	1	1	0	1	5	LOW	II	1
Li 2019	0	1	1	1	1	1	5	LOW	III	2
Noh 2019	0	1	1	1	1	1	5	LOW	III	2
Xu 2019	0	1	1	1	0	1	4	MODERATE	III	3
Brodbelt 2019	0	1	1	1	1	1	5	LOW	II	1
Chin 2019	0	1	1	1	1	1	5	LOW	II	1
Frati 2019	1	1	1	1	1	1	6	LOW	II	1
Maiuri 2019	1	1	1	1	1	1	6	LOW	II	1
Wright 2019	0	1	1	1	1	1	5	LOW	II	1
Yeo 2019	1	1	1	1	1	1	6	LOW	II	1
Yolcu 2019	0	1	1	1	1	1	5	LOW	II	1
Elkatatny 2019	1	1	1	0	0	1	4	MODERATE	II	2
Gilard 2018	0	1	1	1	1	1	5	LOW	II	1
Hua 2018	1	1	1	1	1	1	6	LOW	II	1
Iwata 2018	1	1	1	1	1	1	6	LOW	II	1
Santos 2018	1	1	1	1	1	1	6	LOW	II	1
Schwake 2018	1	1	1	1	0	1	5	LOW	II	1
Takashima 2018	1	1	1	1	1	1	6	LOW	II	1
Onken 2018	0	1	1	1	0	1	4	MODERATE	II	2
Harel 2017	0	1	1	1	0	1	4	MODERATE	III	3
Notani 2017	0	1	1	1	1	1	5	LOW	III	2

Wu 2017	0	1	1	1	1	1	5	LOW	III	2
Bayoumi 2017	1	1	1	1	1	1	6	LOW	II	1
Davies 2017	1	1	1	1	1	1	6	LOW	II	1
Raco 2017	1	1	1	1	1	1	6	LOW	II	1
Tola 2016	0	1	1	1	1	1	5	LOW	III	2
Ye 2016	0	1	1	1	1	1	5	LOW	III	2
Kim 2016	0	1	1	1	1	1	5	LOW	II	1
Lonjon 2016	0	1	1	1	1	1	5	LOW	II	1
Maiti 2016	1	1	1	1	1	1	6	LOW	II	1
Pompili 2016	0	1	1	1	1	1	5	LOW	II	1
Zham 2016	1	1	1	1	0	1	5	LOW	II	1
Viereck 2016	0	1	1	1	0	1	4	MODERATE	II	2
Aboul-Enein 2015	0	1	1	1	1	1	5	LOW	III	2
Lee 2015	0	1	1	1	1	1	5	LOW	III	2
Sun 2015	0	1	1	1	1	1	5	LOW	II	1
Golanov 2015	0	1	1	1	0	1	4	MODERATE	II	2
Haq 2015	0	1	1	1	0	1	4	MODERATE	II	2
Turel 2015	0	1	1	1	0	1	4	MODERATE	II	2
Tsuda 2014	1	1	1	1	0	1	5	LOW	III	2
Ambekar 2014	1	1	1	1	1	1	6	LOW	II	1
Arima 2014	0	1	1	1	1	1	5	LOW	II	1

Gu 2014	1	1	1	1	1	1	6	LOW	II	1
Riad 2013	1	1	1	1	1	1	6	LOW	II	1
Barresi 2012	1	1	1	1	1	1	6	LOW	III	2
Wang 2012	0	1	1	1	1	1	5	LOW	III	2
Iacoangeli 2012	0	1	1	1	1	1	5	LOW	II	1
Nakamura 2012	1	1	1	1	0	1	5	LOW	II	1
Kufeld 2012	0	1	1	1	0	1	4	MODERATE	II	2
Maiuri 2011	1	1	1	1	1	1	6	LOW	II	1
Postalci 2011	1	1	1	1	1	1	6	LOW	II	1
Voulgaris 2010	0	1	1	1	1	1	5	LOW	III	2
Engelhard 2010	0	1	1	1	1	1	5	LOW	II	1
Liu 2009	1	1	1	1	1	1	6	LOW	II	1
Sacko 2009	0	1	1	1	1	1	5	LOW	II	1
Boström 2008	1	1	1	1	1	1	6	LOW	II	1
Sandalcioglu 2008	1	1	1	1	1	1	6	LOW	II	1
Schröder 2008	1	1	1	1	1	1	6	LOW	II	1
Gerszten 2008	0	1	1	1	0	1	4	MODERATE	II	2
Setzer 2007	1	1	1	1	1	1	6	LOW	II	1
Yoon 2007	1	1	1	1	0	1	5	LOW	II	1

Lee 2006	1	1	1	1	1	1	6	LOW	II	1
Sayagués 2006	1	1	1	1	1	1	6	LOW	II	1
De Verdelhan 2005	1	1	1	1	1	1	6	LOW	III	2
Haegelen 2005	0	1	1	1	1	1	5	LOW	III	2
Peker 2005	1	1	1	1	0	1	5	LOW	II	1
Schaller 2005	1	1	1	1	1	1	6	LOW	II	1
Morandi 2004	0	1	1	1	1	1	5	LOW	III	2
Arslantas 2003	0	1	1	1	1	1	5	LOW	III	2
Gambardella 2003	0	1	1	1	1	1	5	LOW	III	2
Cohen-Gadol 2003	1	1	1	1	1	1	6	LOW	II	1
Schick 2001	0	1	1	1	1	1	5	LOW	II	1
Gezen 2000	1	1	1	1	1	1	6	LOW	II	1

ROB = Risk of Bias, OCEBM = Oxford Center of Evidence Based Medicine

Table S3. Epidemiology

Study ID	Spinal meningioma patients	Females	Males	% Females	M:F	Mean age
Champeaux-Depond 2022	2844	2251	593	79.1%	1:3.8	66
Gheorghiu 2022	237	207	30	87.3%	1:6.9	NM
Aoyama 2021	15	9	6	60%	1:1.5	62.7
Cao 2021	4204	3367	837	80.1%	1:4	62.86
Pettersson-Segerlind 2021	129	106	23	82.2%	1:4.6	65
Baro 2021	90	75	15	83.3%	1:5	67
Colli 2021	37	31	6	83.8%	1:5.2	NM
Corell 2021	111	86	25	77.5%	1:3.4	62.5
Davarski 2021	31	29	2	93.5%	1:14.5	65
Kilinc 2021	119	84	35	70.6%	1:2.4	59.9
Kobayashi 2021	53	42	11	79.2%	1:3.8	62.4
Kobayashi 2021	116	94	22	81%	1:4.3	61.2
Ono H. 2021	14	12	2	85.7%	1:6	67.9
Ono K. 2021	24	20	4	83.3%	1:5	65.4
Tominaga 2021	29	22	7	75.9%	1:3.1	NM
Wach 2021	123	94	29	76.4%	1:3.2	65.6
Han 2020	336	259	77	77.1%	1:3.4	49.9
Hohenberger 2020	45	39	6	86.7%	1:6.5	63
Kwee 2020	166	139	27	83.7%	1:5.1	66
Lee 2020	59	49	10	83.1%	1:4.9	59.7
Portet 2020	30	27	3	90%	1:9	73
Voldřich 2020	84	68	16	81%	1:4.3	65
Yamaguchi 2020	53	48	5	90.6%	1:9.6	60
Elkatatny 2019	45	33	12	73.3%	1:2.8	42
Yeo 2019	105	92	13	87.6%	1:7.1	61.5
Gilard 2018	87	70	17	80.5%	1:4.1	64.6
Hua 2018	483	384	99	79.5%	1:3.9	53.76
Iwata 2018	24	16	8	66.7%	1:2	68
Onken 2018	210	170	40	81%	1:4.3	65
Santos 2018	51	40	11	78.4%	1:3.6	57.6
Schwake 2018	88	74	14	84.1%	1:5.3	

Takashima 2018	20	16	4	80%	1:4	63.3
Davies 2017	31	27	4	87.1%	1:6.8	64
Wu 2017	14	5	9	35.7%	1:0.6	11.1
Kim 2016	20	15	5	75%	1:3	59
Maiti 2016	38	31	7	81.6%	1:4.4	56
Pompili 2016	27	25	2	92.6%	1:12.5	59.5
Tola 2016	20	14	6	70%	1:2.3	61
Zham 2016	39	25	14	64.1%	1:1.8	51.6
Tsuda 2014	14	11	3	78.6%	1:3.7	56.2
Ambekar 2014	13698	9160	4538	66.90%	1:2	NM
Gu 2014	31	19	12	61.3%	1:1.6	NM
Riad 2013	15	13	2	86.7%	1:6.5	67.6
Barresi 2012	58	48	10	82.8%	1:4.8	59.1
Nakamura 2012	68	56	12	82.4%	1:4.7	56
Iacoangeli 2012	30	20	10	66.7%	1:2	74.6
Wang 2012	10	2	8	20%	1:0.3	13.2
Maiuri 2011	117	87	30	74.4%	1:2.9	59
Postalci 2011	46	33	13	71.7%	1:2.5	52
Engelhard 2010	105	90	15	85.7%	1:6	NM
Liu 2009	36	29	7	80.6%	1:4.1	NM
Sacko 2009	102	87	15	85.3%	1:5.8	74.6
Boström 2008	61	50	11	82%	1:4.5	61
Sandalcioglu 2008	131	114	17	87%	1:6.7	69
Schröder 2008	30	23	7	76.7%	1:3.3	68
Setzer 2007	80	58	22	72.5%	1:2.6	61.9
Yoon 2007	38	31	7	81.6%	1:4.4	52
Sayagués 2006	14	13	1	92.9%	1:13	64
De Verdelhan 2005	23	19	4	82.6%	1:4.8	60
Peker 2005	41	32	9	78%	1:3.6	50
Schaller 2005	33	30	3	90.9%	1:10	63
Morandi 2004	30	25	5	83.3%	1:5	77.1
Arslantas 2003	16	13	3	81.3%	1:4.3	50.12
Cohen-Gadol 2003	80	68	12	85%	1:5.7	63
Schick 2001	81	NM	NM	>50%	NM	NM
Gezen 2000	36	27	9	75%	1:3	49

NM = Not Mentioned

Table S4. Histopathology

Study ID	All spinal meningiomas	WHO 1	WHO 2	WHO 3	WHO 1 subtypes									WHO 2 subtypes			WHO 3 subtypes			Others
					Meningothelial	Fibroblastic	Transitional	Psammoma	Angiomatous	Microcystic	Secretory	Lymphoplasmacyte-rich	Metaplastic	Chordoid	Clear cell	Atypical	Papillary	Rhabdoid	Anaplastic	
Ampie 2021	46	43	2	1	8		1	11					2			2			1	3 multiple components<br>18 WHO I tumors were of unspecified subtype
Pettersson-Segerlind 2021	129	127	2	0																
Baro 2021	90	85	4	1																
Kwee 2020	166	143	7	0	24		48	71								7				16 tumors of unspecified subtypes
Han 2020	337	317	15	5										2	5	8	1		4	
Schwake 2018	88	86	1	0	49	3	23	9	1				1			1				1 unclear
Maiti 2016	38	35	3	0																
Tsuda 2014	14	14	0	0	4	2	3	3	1	1										
Barresi 2012	58	55	3	0	8	5	26	8	1				7		2	1				
Nakamura 2012	68	67	0	1	23	2	11	22	5				4				1			

Wang 2012	10	7	3	0	1	3		3							1	2				
Maiuri 2011	117	114	2	0	35	5	32	39	1				2			2				1 sclerosing
Yoon 2007	38	36	0	2	16	4	5	7												4 had multiple components 2 were WHO III of unspecified subtype
Cohen-Gadol 2003	80	77	2	0	30	3	9	34					1	1	1					1 unspecified
Gezen 2000	36	36	0	0	10		7	19												
Hohenberger 2020	45	34	1	0	11	2	2	12	1					?	?	?				5 had multiple components 1 WHO II of unspecified subtype 1 sclerosing 10 unknown
Raco 2017	173	170	2	1		23		47								2			1	Of the 170 WHO I tumors, the

																				subtype of only 88 was determined, 18 of which had multiple components
Schaller 2005	33	33	0	0	4	7		22												
Cao 2021	1483	1388	95	1	378	43	166	786	15						41	53	1			
Colli 2021	36	36	0	0	8	2	16	9					1							
Hua 2018	483	461	14	8	128	180	38	95	6	1	2	4	7		6	8			8	
Voulgaris 2010	10	10	0	0	2		3	5												
Maiuri 2019	28	26	2	0	2	1	8	13	2							2				
Portet 2020	30	30	0	0	13		6	11												
Kobayashi 2021	116	113	3	0	71	11	7	24								3				
Lonjon 2016	23	21	1	0	10		1	8												2 multiple components<br>1 WHO II psammoma<br>1 missing
Kilinc 2021	119	112	7	0	93	3	4	9	1					?	?	?				2 unknown<br>7 WHOII of

																				unspecified histopath
Setzer 2007	80	69	7	2	46	2	7	13		1				1		6				2 WHO IV meningiosarcomas terminology no longer in use
Gilard 2018	87	78	3	0	34	6	8	26	4					?	?	?				3 WHO II tumors of unspecified subtype 6 unknown
Boström 2008	61	61	0	0	19	5	3	32	2											
Davarski 2021	31	29	2	0				29								2				
Riad 2013	15	15	0	0	8			7												
Santos 2018	51	48	3	0	6		25	17								3				
Tola 2016	20	20	0	0	7	1		12												
Yeo 2019	105	102	3	0	40	2	14	44		1			1			3				
Zham 2016	39	34	5	0	7	3	9	15						2	3					
Wright 2019	287	0	211	76																
Noh 2019	13	0	13	0												17				
Wach 2021	123	114	9	0																
Sun 2015	20	0	20	0										2	2	16				
Corell 2021	111	106	5	0																

Davies 2017	31	29	2	0																
Onken 2018	207	201	6	0																
Postalci 2011	46	44	2	0																
Sandalciogl u 2008	131	129	2	0																
Yamaguchi 2020	53	50	3	0											1	2				
Ye 2016	25	0	20	5										1	2	17	3		2	
Wu 2017	14	9	5	0	4		2	3						1	3	1				
Lee 2006	75	75	0	0	60															15 non-meningoth elial tumors of unspecifie d subtype.
Krauss 2021	189	172	15	2											3	9	1		1	2 had multiple componen ts<br>1 WHO II fibrous<br>172 WHO I tumors of unspecifie d subtype
Sayagués 2006	14	14	0	0	5		3	6												
Arslantas 2003	16	15	1	0	1		13	1								1				
Li 2019	12	0	12	0											12					

Xu 2019	17	17	0	0	5		4	7	1											
Kobayashi 2021	53	53	0	0	35	1	4	13												
Ono K. 2021	22	21	1	0	10		5	6							1					
Aoyama 2021	15	15	0	0	8	1		5					1							
Kim 2016	20	19	1	0	13	1	4		1						1					
Tominaga 2021	29	29	0	0	13	1	2	13												
Peker 2005	41	38	0	0	17	3	8	10												3 unknown
Schröder 2008	30	23	1	0	17			6												6 unknown
Elkatatny 2019	45	39	6	0	15	6	9	9								6				
Iacoangeli 2012	65	65	0	0	37	10	4	14												
Morandi 2004	30	27	3	0	4	1	7	15								3				
Saiwai 2021	39	39	0	0	19	3	3	14												

WHO = World Health Organization

Table S5. Genetics and immunohistochemistry

Study ID	mean Ki-67/MIB-1 (%)	Range Ki-67/MIB-1	ER+/-	PR +/-	AR +/-	# NF2	Other syndromes
Champeaux-Depond 2022						25	
Pettersson-Segerlind 2021	Median: 4.5	0-20				1	
Ono K. 2021	2.4						
Wach 2021	Median: 4	2-12					
Ampie 2021						3	1 VHL
Baro 2021						2	
Portet 2020			9/21	29/1	30/0		
Telugu 2020				7/4			
Pemov 2020						4	
Kwee 2020						2	
Han 2020						1	
Voldřich 2020						2	
Hohenberger 2020						14*	
Li 2019				7/1			
Noh 2019	5.7						
Maiuri 2019							
Hua 2018	1.94	1–20		74/120			
Schwake 2018						1	
Wu 2017	1.6	1-4					

Raco 2017						0	
Maiti 2016						6	
Sun 2015	11.2	5.8-23.8					
Tsuda 2014						1	
Barresi 2012	1.4	1-5	0/58	50/8		2	
Wang 2012	1.5	1-3	0/10	6/4		4	
Nakamura 2012						2	
Maiuri 2011			0/20	20/0		3	
Voulgaris 2010	2.75	0.5-7					
Yoon 2007						1	1 NF1
Cohen-Gadol 2003						5	
Gezen 2000						1	

**Not specified whether NF1 or NF2, NF = Neurofibromatosis, VHL = von Hippel-Lindau, AR = Androgen Receptor, ER = Estrogen receptor, PR = Progesterone Receptor*

Table S6. Tumor location

Study ID	Number of spinal meningiomas	Cer	CT	Th	TL	L	Sacral	CCJ (excluded)	D	V	L	DL	VL	Circumferent	Dumbbell shape
Davarski 2021	31	5	2	21	2	1			7	2	1	14	7		2
Corell 2021	108	26		70		11	1	9							
Pettersson-Segerlind 2021	129	39		89		1									
Kilinc 2021	119	29	4	82	1	3			2	8	73	9	24		
Kobayashi 2021	116	22		90		4			10	34	64				3
Wach 2021	123	31		90		2			36	37	49				
Ampie 2021	74	22		50		2			31/46	15/46					
Ono K. 2021	22	6		17		1			8	10	5			1	
Baro 2021	90	13	2	73	1	1			22	7	34	5	22		
Ono H. 2021	14	5		9											
Aoyama 2021	15	6		9											
Kobayashi 2021	53	14		38		1			10	16	27				
Saiwai 2021	39	6		32					7	4	27				
Tominaga 2021	29	5		23		1			5	2		5	17		
Colli 2021	36	9	3	23	2										
Voldřich 2020	84	15		69											4
Han 2020	337	115	12	174	12	24				8/19		6/19		6/19	5/19
Hohenberger 2020	45	19		25		1			2	5	32	1	5		

Yamaguchi 2020	40	6		34				13	4	2	17	14	16		
Kwee 2020	161	27	4	118	4	8		5	21/151	35/151	95/151				
Naito 2020	23	4		17		2		12	18	17					2
Lee 2020	59	12		44		3									2
Maiuri 2020	56	13		42		1			2	3	9	20	22		
Li 2019	12	2		1		7	2								
Yeo 2019	105	38		64		3			2	10	43	21	29		5
Chin 2019	39	16		18		5									
Noh 2019	17	3		9			1			2		4	7		
Elkatatny 2019	45	12		27		6									
Xu 2019	17		1	16											
Santos 2018	51	15	4	32											
Hua 2018	194	57	5	125	4	3			25	47	122				
Schwake 2018	88	22		64		2									3
Gilard 2018	87	22		57		7	1		19	15	53				
Takashima 2018	20	8		11		1									
Onken 2018	207								76	48	86				
Raco 2017	173	25	27	119	2				11	15	32	42	73		4
Wu 2017	14	7		5		2									4
Davies 2017	31	3	1	27											
Notani 2017	12	3		9						4			8		
Pompili 2016	29	5		21		3									

Lonjon 2016	23	23													
Ye 2016	25	10		10		5				4		21			
Tola 2016	20	3		16		1			2	4	3		11		
Maiti 2016	38	10	4	24						2	20		12		2
Kim 2016	20	8		12					2	9	9				
Sun 2015	20	7		11	2					9		11			
Haq 2015	48	8	3	35	1	1									2
Aboul-Enein 2015	16	16								4	1		11		
Golanov 2015	27	16		11											2
Lee 2015	10	5		4		1		1		2	1	1	4	3	1
Tsuda 2014	14	4		10					7	4	3				
Arima 2014	13	4		9				10	8	15					2
Gu 2014	31	5		7		19									
Riad 2013	15	2		11		2			1		3	4	7		
Iacoangeli 2012	65	12		53											
Wang 2012	10	5	2	3											2?
Nakamura 2012	68	14		50		4			11	38	15				4
Barresi 2012	58	6		48		4									
Postalci 2011	46	4		39		3			30	7		9			2
Maiuri 2011	117	25		90		2			17	9	91				
Voulgaris 2010	10			9		1				10					
Sacko 2009	102	11	4	87					26	13	63				1
Liu 2009	36	5		31											

Boström 2008	61	11		51							36	9	12	1	
Sandalcioglu 2008	131	21	7	95	6	2			13	12	46	22	38		
Schröder 2008	22	1	6	9	5	1		8							
Gerszten 2008	13	10		2			1								
Setzer 2007	80	17	6	48	6	3			5	14	11	17	33		5
Yoon 2007	38	6	2	28	1	1			1/30	5/30	12/30	10/30			2/30
Schaller 2005	33	10		23					8	6	19				2
Peker 2005	41	7		34											
De Verdelhan 2005	24	3		21					2	3	7	7	5		
Haegelen 2005	56	13		42		1			2	3	9	20	22		
Morandi 2004	30	2		28											1
Cohen-Gadol 2003	80	24		55		2			2/41	3/41	11/41	12/41	10/41	3/41	10/71
Gambardella 2003	10			10						10					
Arslantas 2003	16	3		13											
Gezen 2000	36	5	2	20	4	5			12	7	18				

Cer = Cervical, CT = Cervicothoracal, Th = thoracal, TL, Thoracolumbar, L = Lumbar, V = Ventral , VL = Ventrolateral, D = Dorsal, DL = Dorsolateral, L = Lateral,

Table S7. Presenting symptoms

Study ID	Most common presenting symptom	Second most common	Third most common	Asymptomatic	Mean symptom duration (mos)
Champeaux-Depond 2022	motor dysfunction	bladder or bowel dysfunction	gait disturbance		
Ampie 2021	sensory dysfunction<br>motor dysfunction			2	
Corell 2021	sensory dysfunction	motor dysfunction	gait disturbance	6	
Kilinc 2021	sensory dysfunction	gait disturbance	motor dysfunction	3	
Wach 2021	pain	sensory dysfunction	motor dysfunction	4	
Pettersson-Segerlind 2021	motor dysfunction	sensory dysfunction	gait disturbance		
Davarski 2021	motor dysfunction	pain	bladder or bowel dysfunction		6.45
Kobayashi 2021	gait disturbance	pain	motor dysfunction	9	11.3
Baro 2021					median: 9
Ono K. 2021					17.5
Kobayashi 2021					10.8
Ono H. 2021					10
Hohenberger 2020	sensory dysfunction	gait disturbance	motor dysfunction		3.6
Han 2020	pain	sensory dysfunction<br>motor dysfunction	bladder or bowel dysfunction		15.29
Yamaguchi 2020	pain	sensory dysfunction	motor dysfunction		
Kwee 2020	motor dysfunction	sensory dysfunction	pain		

Voldřich 2020	motor dysfunction	sensory dysfunction	bladder or bowel dysfunction	10	10.4
Noh 2019	sensory dysfunction	motor dysfunction<br>gait disturbance	pain		7.7
Yeo 2019	sensory and motor dysfunction	pain	gait disturbance	4	
Chin 2019	pain	sensory dysfunction	motor dysfunction	12	
Li 2019	pain	sensory and motor dysfunction	bladder or bowel dysfunction		27.6
Elkatatny 2019	motor dysfunction	bladder or bowel dysfunction and pain			9.5
Xu 2019					19.3
Santos 2018	sensory dysfunction<br>motor dysfunction	pain	bladder or bowel dysfunction		20
Hua 2018	sensory dysfunction	pain	motor dysfunction	26	14.91
Schwake 2018	sensory dysfunction	motor dysfunction	pain		
Gilard 2018	motor dysfunction	pain (back)		6	5.4
Onken 2018					0.25-3 (25%), 3-12 (56%), and > 12 (18%)
Davies 2017	sensory dysfunction	motor dysfunction			
Raco 2017	pain	motor and gait dysfunction	sensory dysfunction		20.01
Wu 2017	motor dysfunction	pain	sensory dysfunction		9.7
Ye 2016	sensory dysfunction	motor dysfunction	pain		
Pompili 2016	pain	motor dysfunction	bladder or bowel dysfunction		

Tola 2016	pain	gait disturbance	sensory dysfunction		6
Lonjon 2016	motor dysfunction	gait disturbance	pain		
Sun 2015	pain<br>sensory dysfunction	motor dysfunction	gait disturbance	1	
Haq 2015	pain	sensory dysfunction	motor dysfunction		
Aboul-Enein 2015	pain	sensory dysfunction	motor dysfunction		
Tsuda 2014	sensory dysfunction	motor dysfunction	pain		
Riad 2013	gait disturbance	sensory dysfunction<br>motor dysfunction	bladder or bowel dysfunction<br>pain		11
Iacoangeli 2012	pain (back)	motor dysfunction	bladder or bowel dysfunction		
Wang 2012	pain	motor dysfunction	gait disturbance		5.7
Postalci 2011	pain	sensory and motor dysfunction	bladder or bowel dysfunction	3	
Engelhard 2010	motor dysfunction	sensory dysfunction	gait disturbance		
Voulgaris 2010	motor dysfunction	pain	gait disturbance		
Sacko 2009	bladder or bowel dysfunction	pain	sensory dysfunction		9.5
Boström 2008	sensory dysfunction	gait disturbance	motor dysfunction		
Sandalcioglu 2008	sensory and motor dysfunction	gait disturbance	pain		
Schröder 2008	motor dysfunction	gait disturbance	pain<br>sensory dysfunction		12
Setzer 2007	sensory dysfunction	gait disturbance	motor dysfunction		11.8
Yoon 2007	motor dysfunction				12

Schaller 2005	pain	sensory dysfunction	bladder or bowel dysfunction		22
Peker 2005	pain	motor dysfunction	sensory dysfunction		14
Morandi 2004					13.4
Cohen-Gadol 2003	sensory dysfunction	gait disturbance	pain		
Gambardella 2003	pain<br>motor dysfunction	sensory dysfunction			25.2
Gezen 2000	pain<br>motor dysfunction	sensory dysfunction	bladder or bowel dysfunction		

Table S8. Non-surgical treatment options

Study ID	Type of non-surgical treatment option	Number of patients	Number of tumors treated with Primary/Adjuvant/Salvage therapies	% with non-surgical treatment options	Doses	Indications	Outcomes and complications of the therapy
Ampie 2021	Radiotherapy	2	0/2/0	4.3%	31.8 Gy as reported for only 1 of the patients	WHO grade III and subtotal resection	Radiotherapy and RTK-inhibitors did not benefit the patient with WHO grade III tumor as he died shortly after the therapy
Pettersson-Segerlind 2021	Radiotherapy	1	0/1/0	0.8%		Subtotal resection (however only 1 of 20 subtotal resected spinal meningiomas received further adjuvant radiotherapy)	
Krauss 2021	Radiotherapy	10	0/7/3	23.5%	- 33x180 cGy<br>- 30x180 cGy<br>- 30x180 cGy<br>- 35 fractions<br>- 33x180 cGy<br>- 33x180 cGy<br>- 28x180 cGy<br>- 28x180 cGy<br>- Not stated<br>- 5600 cGy	WHO grade II and III spinal meningiomas and recurrent tumors	Two patient who received adjuvant radiotherapy experienced recurrence.<br>Adjuvant radiotherapy was associated with significantly lower rates of recurrence.<br>Complications were reported in 3 patients: radiation necrosis, panic attack, and constipation following radiation therapy.
Kobayashi 2021	Radiotherapy	2	0/2/0	2%	- 48Gy<br>- 54Gy	WHO grade II	Both patients experienced recurrence even with radiotherapy
Cao 2021	Radiotherapy	108	Not mentioned	2.6%			Patients who received adjuvant radiotherapy usually had worse

							survival outcomes than those who did not (significant on univariate but not on multivariate analysis)
Kwee 2020	Radiotherapy	1	0/0/1	0.6%		Recurrent WHO grade II tumor (however only 1/6 recurrent WHO grade II received radiotherapy)	
Han 2020	Radiotherapy	1	0/1/0	5.3%		High grade (WHO II and III) spinal meningiomas	
Yolcu 2019	Stereotactic radiosurgery (n=111) Radiotherapy (n=156) Bradytherapy (n=1)	268	131/137/0	100%	Mean radiation dose for stereotactic radiosurgery: 24Gy (8-200Gy) Mean radiation dose for radiotherapy: 40.4Gy (2.5-540Gy)	Multivariate analysis revealed that radiation-based therapies were most commonly used for patients with subtotal resection or those with higher WHO grade tumors	Significantly worse survival outcomes for WHO II & III tumors who received radiation than those who did not
Chin 2019	Stereotactic radiosurgery	39	20/0/19	100%	Median prescription dose: 20Gy (16-30Gy) Median number of fractions: 2 (1-3)	Neurofibromatosis constituted one of the indications	Five spinal meningiomas recurred after stereotactic radiosurgery, 4 of which occurred in the same patient (median follow-up: 46 months). 10/16 patients that initially presented with pain had either improved or stabilized and 6/16 were lost to follow-up.
Li 2019	None	0		0%			42% of patients presented with recurrences

Schwake 2018	Radiotherapy	1	0/0/1	1.1%		Surgery contraindicated as the patient experience a 3rd recurrence after 2 separate resection surgeries	
Wu 2017	Radiotherapy	3	0/0/3	21.4%		Recurrent tumors	
Raco 2017	None	0		0%			
Lonjon 2016	Radiotherapy (n=4), Stereotactic radiosurgery (n=1)	5	0/3/2	21%	- 24Gy (SRS)<br>- 50Gy<br>- 50Gy<br>- 50Gy<br>- 65Gy	Recurrent tumors; residual tumor around the vertebral artery; and WHO grade II	Further recurrence was observed in 2/5, both had received radiotherapy.
Ye 2016	Radiotherapy	1	0/1/0	4%		Simpson grade 2 resection	The patient experienced local recurrence and died
Golanov 2015	Stereotactic radiosurgery	27	17/0/10	100%	Mean dose per fraction: 15.9 Gy (14.1-16.2 Gy)	Minimal to no neurological symptoms associated with tumor; Remnant/recurrent tumor or continued tumor growth after surgery; Contraindications for surgery	Total or partial tumor control was achieved in all patients and none of the patients experienced continued growth (mean follow-up: 18.6 months)<br>All patients had either stable or improved neurological status after radiosurgery
Lee 2015	Stereotactic radiosurgery	11	6/4/1	100%	Median prescribed dose: 26 Gy (22–30 Gy)<br>Median number of fractions: 3 (2-4)		Tumor control was achieved in all patients (mean follow-up: 46.9 months).<br>One patient experienced radiation-induced cord toxicity.<br>T2-signal intensity either regressed

							or stabilized in most patients after the procedure.
Aboul-Enein 2015	Radiotherapy	1	0/1/0	6.2%		Atypical (WHO II) spinal meningioma	The patient experienced recurrence even through both surgery and adjuvant radiation
Sun 2015	Radiotherapy	2	0/2/0	10%	- 1.8Gy<br>- 54.8Gy	WHO grade II spinal meningiomas	One patient had to stop early on due to complication in the form of worsening lower extremity sensory loss and ataxia while the other sustained arachnoiditis 6 months after radiotherapy
Tsuda 2014	Radiotherapy	1	0/1/0	7.1%	50Gy	High MIB-1 labeling index (8%) and Subtotal resection (Simpson grade 4)	
Riad 2013	None	0		0%			
Kufeld 2012	Stereotactic radiosurgery	11	4/3/4	100%	Median prescription dose: 14 Gy (13-15Gy)	Remnant/recurrent or multiple spinal meningiomas	There were no recurrences (median follow-up: 18 months).<br>In most patients either improvement or stabilization of clinical status were achieved. Only one patient experienced transient neurological worsening.<br>Some patients experienced nausea after therapy.
Wang 2012	Radiotherapy	1	0/1/0	10%		Radiotherapy was considered in cases of higher WHO grades after incomplete removal or recurrence	

						in children who were more than 5 years old	
Postalci 2011	None	0		0%			
Engelhard 2010	Radiotherapy	1	0/1/0	1%			
Gerszten 2008	Stereotactic radiosurgery	13	2/2/11	100%	Mean maximum tumor dose (Dmax): 2125Gy (1750–2500Gy)	Patients for whom microsurgical resection was contraindicated, tumors that recurred, underlying neurofibromatosis (NF) with multiple lesions, or strong patient preferences	Tumor control was achieved in all patients (median follow-up: 37 months).<br>One patient experienced radiation-induced spinal cord toxicity.
Schröder 2008	Radiotherapy	1	0/1/0	3.3%		Atypical (WHO II) spinal meningiomas	
Yoon 2007	Radiotherapy	4	0/4/0	10.5%		Inoperable tumors with high risk of complication, higher WHO grade meningiomas and with subtotal resection (Simpson grade 4)	
Setzer 2007	Chemotherapy (n=1), Radiotherapy (n=2), Combined	5	0/5/0	6.3%			

	chemoradiotherapy (n=1), Stereotactic radiosurgery (n=1)						
Schaller 2005	Radiotherapy	5	0/5/0	15.2%			There were neither any long-term side effects of radiation therapy nor any recurrence of the tumors.
Gezen 2000	Radiotherapy	2	0/2/0	5.6%		Tumor recurrence	No further recurrence observed.

Table S9. Surgical treatment of spinal meningniomas

Study ID	Number of tumors operated	Surgical classification (Simpson, Saito, or arbitrary)	Simpson grade 1	Simpson grade 2	Simpson grade 3	Simpson grade 4	Simpson grade 5	Mean duration of surgery (mins)
Ampie 2021	46	Simpson	10.87%	43.48%	28.26%	17.39%	0.00%	
Aoyama 2021	15	Simpson	0.00%	100.00%	0.00%	0.00%	0.00%	
Baro 2021	90	Simpson	8.89%	77.78%	11.11%	2.22%	0.00%	
Corell 2021	111	Simpson	0.00%	100.00%	0.00%	0.00%	0.00%	
Davarski 2021	31	Simpson	93.55%		3.23%	3.23%	0.00%	
Kilinc 2021	119	Simpson	86.55%		0.00%	13.45%		185
Kobayashi 2021	116	Simpson	25.00%	68.10%	3.45%	3.45%	0.00%	
Kobayashi 2021	53	Gross total resection in 100%	n/a	n/a	n/a	n/a	n/a	214 ± 68 (longer in calcified tumors, but not significant)
Krauss 2021	17	Simpson (4 had unknown resection grade)	5.88%	11.76%	41.18%	17.65%	0.00%	
Ono K. 2021	24	Simpson	70.83%	29.17%	0.00%	0.00%	0.00%	
Pettersson-Segerlind 2021	129	Simpson	0.00%	71.32%	13.18%	15.50%	0.00%	
Saiwai 2021	38	Simpson grade 2 (group 1, n=26) vs. Saito (group 2, n=12)	n/a	n/a	n/a	n/a	n/a	
Tominaga 2021	29	Simpson (group 1, n=19)	n/a	n/a	n/a	n/a	n/a	

		vs.<br>Saito (group 2, n=10)						
Wach 2021	123	Simpson	47.97%	47.97%	2.44%	1.63%	0.00%	Median: 178.9 (range: 130.0–204.0)
Han 2020	20	Simpson	0.00%	80.00%	20.00%	0.00%	0.00%	
Hohenberger 2020	45	Simpson	13.33%	80.00%	6.67%	0.00%	0.00%	233 (range: 106–362)
Kwee 2020	159	Simpson	20.75%	60.38%	7.55%	11.32%	0.00%	
Naito 2020	70	Simpson	14.29%	74.29%	0.00%	11.43%	0.00%	
Voldřich 2020	92	Simpson	0.00%	91.30%	8.70%	0.00%	0.00%	
Elkatatny 2019	45	Total in 86.7%<br>Subtotal in 13.3%	n/a	n/a	n/a	n/a	n/a	
Li 2019	12	Simpson	75.00%		25.00%		0.00%	
Noh 2019	13	Simpson	30.77%	69.23%	0.00%	0.00%	0.00%	
Xu 2019	17	Not mentioned	n/a	n/a	n/a	n/a	n/a	153.2 (range: 115-300)
Gilard 2018	87	Simpson	5.75%	87.36%	5.75%	1.15%	0.00%	
Hua 2018	194	Simpson	30.93%	65.46%	3.61%	0.00%	0.00%	
Onken 2018	207	Simpson	n/a	>90%	n/a	n/a	n/a	Through unilateral posterior approach (with hemilaminectomy):<br>- 136 for anterior tumors<br>- 131 for posterior ones<br>Through bilateral posterior approach (with laminectomy or laminotomy):<br>- 224 for anterior tumors<br>- 148 for posterior ones.
Santos 2018	51	Simpson	41.18%	45.10%	7.84%	5.88%	0.00%	

Schwake 2018	84	Simpson	14.29%	64.29%	16.67%	4.76%	0.00%	
Bayoumi 2017	58	Simpson	98.28%			0.00%	1.72%	
Notani 2017	12	Simpson	16.67%	83.33%	0.00%	0.00%	0.00%	218 (range: 115–315)
Raco 2017	173	Simpson	30.06%	68.79%	1.16%	0.00%	0.00%	
Wu 2017	14	Gross total resection in 78.5%<br>Subtotal resection in 14.2%<br>Partial resection in 7.3%	n/a	n/a	n/a	n/a	n/a	
Kim 2016	73	Simpson	28.77%	36.99%	23.29%	10.96%	0.00%	
Lonjon 2016	23	Simpson	21.74%	39.13%	13.04%	21.74%	4.35%	
Maiti 2016	37	Simpson	5.41%	94.59%	0.00%	0.00%	0.00%	
Pompili 2016	29	Simpson	34.48%	65.52%	0.00%	0.00%	0.00%	160 (range: 100-320) for both spinal schwannoma and meningioma surgery
Tola 2016	20	Simpson	90.00%		5.00%	5.00%	0.00%	180 min (SD, 94; range: 90-433) with the longest occuring in calcified meningiomas
Ye 2016	25	Simpson	16.00%	52.00%	16.00%	16.00%	0.00%	
Zham 2016	39	Complete resection ≈ 50%<br>Incomplete resection ≈ 50%	n/a	n/a	n/a	n/a	n/a	
Aboul-Enein 2015	16	Simpson grade 2 (n=11)<br>Saito method (n=4)<br>Subtotal resection (n=1)	n/a	n/a	n/a	n/a	n/a	

Haq 2015	48	Simpson	0.00%	83.33%	12.50%	4.17%	0.00%		
Sun 2015	20	Simpson	15.00%	65.00%	10.00%	10.00%	0.00%		
Arima 2014	23	Simpson	21.74%	56.52%	8.70%	13.04%	0.00%		
Tsuda 2014	13	Simpson	15.38%	61.54%	0.00%	23.08%	0.00%		
Riad 2013	15	Simpson	13.33%	86.67%	0.00%	0.00%	0.00%		
Barresi 2012	58	Simpson	17.24%	63.79%	18.97%	0.00%	0.00%		
Iacoangeli 2012	65	Saito method (group 1, n=30)<br>vs.<br>Simpson grade 1 or 2 (group 2, n=35)	n/a	n/a	n/a	n/a	n/a		group 1 (hemilaminectomy + Saito method): 145 min<br>group 2 (laminectomy + Simpson grade 1 or 2): 171 min
Nakamura 2012	68	Simpson	63.24%	27.94%	4.41%	4.41%	0.00%		
Wang 2012	10	Simpson	60.00%	20.00%	10.00%	10.00%	0.00%		
Maiuri 2011	117	Simpson	0.00%	94.87%	5.13%				
Postalci 2011	35	Simpson	65.71%	11.43%	14.29%	8.57%	0.00%		
Sacko 2009	102	Total in 91%<br>Subtotal in 9%	n/a	n/a	n/a	n/a	n/a		
Boström 2008	61	Simpson	8.20%	91.80%	0.00%	0.00%	0.00%		
Sandalcioglu 2008	131	Simpson	96.95%		3.05%	0.00%	0.00%		
Schröder 2008	30	Total in 90%<br>Subtotal in 10%	n/a	n/a	n/a	n/a	n/a		
Setzer 2007	80	Simpson	5.00%	88.75%	1.25%	5.00%	0.00%		
Yoon 2007	38	Simpson (1 had unknown resection grade)	26.32%	44.74%	10.53%	15.79%	0.00%		

Haegelen 2005	33	Total in 94%<br>Subtotal in 6%	n/a	n/a	n/a	n/a	n/a	
Peker 2005	41	Total in 98%<br>Subtotal in 2%	n/a	n/a	n/a	n/a	n/a	
Schaller 2005	33	Simpson	84.85%		15.15%		0.00%	
Morandi 2004	30	Total in 90%,<br>Subtotal in 10%	n/a	n/a	n/a	n/a	n/a	119 (range: 50–250)
Gambardella 2003	10	Complete vs. incomplete resection (unclear proportions)	n/a	n/a	n/a	n/a	n/a	<240 in all cases
Schick 2001	81	Total in 96.3%<br>Subtotal in 3.7%	n/a	n/a	n/a	n/a	n/a	
Gezen 2000	36	Total in 97%,<br>Subtotal in 3%	n/a	n/a	n/a	n/a	n/a	

Table S10. Intraoperative neuromonitoring

Study ID	Indications for IONM and/or author's opinion	kind of IONM	Frequency of IONM use
Davarski 2021	Meningioma with intramedullary infiltration mainly warranted the use of IONM	not mentioned	3.20%
Ampie 2021	Certain types of IONM signal disruption correlated with postoperative motor deficiencies	not mentioned	78.70%
Kilinc 2021	Not mentioned	Somatosensory and motor-evoked potentials	100%
Baro 2021	May be useful in spinal meningioma resection, but it is not mandatory to achieve a safe resection.	not mentioned	46.70%
Pettersson-Segerlind 2021	IONM was not used due to limited availability.<br>It was also deemed more important in intramedullary rather than extramedullary spinal tumors.	n/a	n/a
Kobayashi 2021	The technology acted as an intraoperative guide for when to pause the surgery and reassess for the appropriate extent of resection.<br>In some cases, deterioration on IOM prompted interruption of the surgery without achievement of the desired extent of resection.	not mentioned	not mentioned
Wach 2021	"Intraoperative neuromonitoring is an essential tool in the modern era of neurosurgery to prevent worsening of neurological outcome during surgery. The present study analyzes a large institutional series between 2000 and 2019. However, we have not included intraoperative neuromonitoring data for analysis due to changes in the medical devices, interobserver bias and incomplete neuromonitoring data"	not mentioned	not mentioned
Kwee 2020	Preliminary positive results were related to the use of IOM; however, the effect sizes were too small to justify its utilization in spinal meningioma surgery.<br>Moreover, it was noted that IOM was used on ventral tumors to a higher extent.	not mentioned	20%

Han 2020	Not mentioned	Somatosensory and motor-evoked potentials	100%
Voldřich 2020	Not mentioned	not mentioned	100%
Hohenberger 2020	IOM whenever feasible should be performed	D-waves, somatosensory-evoked potentials, motor-evoked potentials	44.40%
Elkatatny 2019	The use of IOM could not be associated to improved surgical outcomes or reduced postoperative complications	Somatosensory and motor-evoked potentials	100%
Noh 2019	Not mentioned	Somatosensory and motor-evoked potentials	not mentioned
Xu 2019	Not mentioned	Somatosensory and motor-evoked potentials	100%
Hua 2018	Not mentioned	Somatosensory-evoked potentials	100%
Schwake 2018	Not mentioned	not mentioned	56%
Wu 2017	Not mentioned	Somatosensory and motor-evoked potentials	100%
Notani 2017	Not mentioned	Motor-evoked potentials	100%
Harel 2017	In this series, the rate of neurological deficits in cases with vs. without IONM is virtually the same, which raises questions about the role of IONM in preventing new neurological deficits<br>No hard evidence warrants the use of IONM for the resection of intradural extramedullary tumors, hence prospective randomized trials comparing the results with and without the use of IONM are needed	Transcranial motor-evoked potentials, somatosensory-evoked potential, and free running electromyography	- 100% of study group (41 tumors, with both meningiomas and nerve sheath tumors)<br>- 0% of control group (70 tumors)
Pompili 2016	Stimulation was used to identify the unaffected rootlets in lumbar and cervical lesions. This allowed safer dissection of the tumor from the main root.	Somatosensory and motor-evoked potentials	100%
Lonjon 2016	Not mentioned	Somatosensory and motor-evoked potentials	100%

Tola 2016	IONM prompted a surgical alert when the nerve root was irritated. In such situations, transcranial motor evoked potentials were also used to identify amplitude changes.	not mentioned	100%
Maiti 2016	Complete resection could be achieved without IOM	Somatosensory and/or motor evoked potentials	rarely used
Haq 2015	The authors advocated the use of IONM but do not mention any use of the technology themselves.	n/a	n/a
Aboul-Enein 2015	Surgical manipulations were stopped whenever any major IONM changes were observed	Somatosensory-evoked potentials	100%
Sun 2015	Not mentioned	not mentioned	100%
Turel 2015	Motor evoked potentials were more reliable than somatosensory ones and were hence used in all cases at the authors' institution.	Motor-evoked potentials	100%
Tsuda 2014	IONM can warn surgeons of an impending possibility of permanent damage.	Motor-evoked potentials	not mentioned
Iacoangeli 2012	Not mentioned	Transcranial motor-evoked potentials, somatosensory-evoked potential, and free running electromyography	100%
Voulgaris 2010	Neurophysiology specialist was available during operations	Transcranial motor-evoked potentials, somatosensory-evoked potential, and free running electromyography	100%
Sandalcioglu 2008	Not mentioned	Somatosensory-evoked potentials	100%
Boström 2008	Not mentioned	not mentioned	100%

IONM = Intraoperative neuromonitoring, n/a = Not applicable

Table S11. Perioperative complications

Study ID	Number of tumors operated	Ibanez 1	Ibanez 2	Ibanez 3	Ibanez 4	Nature of complication not disclosed	Complication rate	Complications by order of frequency
Corell 2021	111	16	7	0	0	0	20.72%	CSF leak was the most common complication, the rest was not stated
Ampie 2021	46	9	2	0	0	0	23.91%	1) New neurological deficit<br>2) Pulmonary embolism; Anemia requiring transfusion; Altered mental status; Hematomyelia & hemorrhagic infarct during surgery; CSF leak
Pettersson-Segerlind 2021	129	7	2	1	0	0	7.75%	1) Wound infection<br>2) CSF leak; Kyphosis<br>3) Myocardial infarction; Tethered spinal cord; Pneumonia
Krauss 2021	17	5	1	1	0	0	41.18%	1) CSF leak; Wound infection; Syringomyelia; Meningitis; Neck pain at surgery site; Paraparesis; Cervical deformity
Baro 2021	90	5	7	0	0	0	13.33%	1) CSF leak<br>2) Hemorrhage<br>3) Wound dehiscence
Kobayashi 2021	116	3	3	0	0	0	5.17%	1) CSF leak; Wound infection requiring surgery (simultaneously in all 3 patients)
Kilinc 2021	119	2	9	0	0	0	9.24%	1) CSF leak<br>2) Wound infection<br>3) Postoperative hematoma; Pulmonary embolism

Davarski 2021	31	1	0	0	0	0	3.23%	1) Hemorrhagic stroke that resolved after conservative treatment
Saiwai 2021	38	1	0	0	0	0	2.63%	1) CSF leak
Wach 2021	123	1	11	0	0	0	9.76%	1) CSF leak<br>2) Epidural hematoma<br>3) Wound infection; Epidural abscess;
Kwee 2020	159	30	4	4	3	0	25.79%	1) CSF leakage<br>2) Postoperative wound infection<br>3) Respiratory insufficiency<br>4) Hyperglycemia; Wound-associated pain; Urinary tract infection; Death<br>5) Pulmonary embolus; Ileus; Cardiac decompensation<br>6) Syrinx formation; Myelum edema with hydrocephalus and secondary meningitis; Skin defect requiring grafting; Seizure; Hypertension; Hypotension
Voldřich 2020	92	8	11	0	0	0	20.65%	1) Epidural hematoma; CSF leak<br>2) Wound infection;<br>3) Laminectomy malposition; Spinal kyphosis; Myelodural adhesions
Hohenberger 2020	45	5	2	0	0	0	15.56%	1) New neurological deficit<br>2) CSF leak; and Wound infection both requiring surgery
Han 2020	20	1	0	0	0	0	5.00%	1) Fever (presumed to be from a meningitis)
Elkatatny 2019	45	7	5	0	0	0	26.67%	1) CSF leak; New neurological deficit
Xu 2019	17	2	0	0	0	0	11.76%	1) CSF leak
Li 2019	12	0	0	0	0	0	0.00%	None

Schwake 2018	84	8	3	1	0	0	14.29%	1) CSF leak<br>2) Urinary tract infection; Wound dehiscence; Decompensated heart failure<br>3) Stroke; Pulmonary embolism; Pneumonia
Onken 2018	207	8	2	0	0	0	4.83%	1) Bleeding<br>2) CSF leak<br>3) Wound dehiscence
Santos 2018	51	3	1	1	0	0	9.80%	1) Syringomyelia<br>2) CSF leak; Cerebral thrombosis; Coma
Gilard 2018	87	0	2	0	2	0	4.60%	1) Death from pulmonary embolism or acute coronary syndrome<br>2) Wound infection; and Hematoma both requiring surgery
Hua 2018	194	0	9	0	0	0	4.64%	1) CSF leak<br>2) Hematoma managed surgically
Raco 2017	173	11	4	0	0	0	8.67%	1) Spinal cord iatrogenic injury<br>2) CSF leak<br>2) Spinal epidural hematoma<br>3) Syringomyelia; Adverse reaction to dural sealant
Notani 2017	12	1	0	0	0	0	8.33%	1) CSF leak
Wu 2017	14	0	2	0	0	0	14.29%	1) Intraspinal infection
Maiti 2016	37	4	2	0	0	0	16.22%	1) Wound complication<br>2) CSF leak
Lonjon 2016	23	0	3	0	1	2	26.09%	1) CSF leak; Epidural abscess; Tumor resection revision; Death
Ye 2016	25	0	1	0	0	0	4.00%	1) CSF leak
Kim 2016	73	0	2	0	0	0	2.74%	1) CSF leak

Tola 2016	20	0	0	0	0	0	0.00%	None
Haq 2015	48	8	0	0	0	0	16.67%	1) CSF leak managed conservatively<br>2) Wound infection, Syrinx formation and spinal cord trauma
Aboul-Enein 2015	16	7	1	0	0	0	50.00%	1) New neurological deficits<br>2) CSF leak
Sun 2015	20	1	0	0	0	0	5.00%	1) Iatrogenic anterior spinal cord (T5-T7) infarction
Tsuda 2014	13	0	1	0	0	0	7.69%	1) Reoperation due to tight dural closure
Ambekar 2014	13698	0	0	0	0	891	6.50%	1) Neurologic complications<br>2) Urinary and renal<br>3) Hemorrhages and hematomas<br>4) Pulmonary<br>5) Cardiac<br>6) Thromboembolic<br>7) Deaths
Riad 2013	15	2	1	0	0	0	20.00%	1) Epidural hematoma requiring surgery; Deep vein thrombosis; CSF leak managed pharmacologically
Iacoangeli 2012	65	16	7	0	0	0	35.38%	1) Long-term back pain<br>2) CSF leak<br>3) Instability requiring fixation<br>4) Pulmonary embolism
Postalci 2011	35	4	3	0	0	0	20.00%	1) New (transient) neurological deficit<br>2) CSF leak
Sacko 2009	102	8	1	0	0	0	8.82%	1) Urinary tract infection<br>2) Phlebitis; Pneumonia; Wound infection; CSF leak

Sandalcioglu 2008	131	4	0	0	1	0	3.82%	1) Venous thrombosis<br>2) CSF leak; Wound dehiscence; Death from myocardial infarction
Schröder 2008	30	0	3	0	0	0	10.00%	1) CSF leak<br>2) CSF flow disturbance
Boström 2008	61	0	2	0	0	0	3.28%	1) Pseudomeningocele with CSF leak; and Wound infection both requiring surgery
Yoon 2007	38	2	2	0	0	0	10.53%	1) CSF leak; Syrinx formation and spinal cord trauma
Setzer 2007	80	1	2	0	1	0	5.00%	1) CSF leak<br>2) Wound infection; Death from pulmonary embolism
Haegelen 2005	33	2	0	0	0	0	6.06%	1) Phlebitis; Pneumonia
Morandi 2004	30	1	0	0	0	0	3.33%	1) Pneumonia
Cohen-Gadol 2003	80	6	6	1	1	0	17.50%	1) New neurological deficits<br>2) Pseudomeningocele; CSF leak; Spinal instability<br>3) Wound infection and revision; Hydrocephalus requiring drainage; Death of respiratory failure
Gambardella 2003	10	0	1	0	0	0	10.00%	1) CSF leak
Gezen 2000	36	2	1	0	1	0	11.11%	1) Wound infection<br>2) CSF leak; perioperative death following a pulmonary embolism

Table S12. Neurological outcomes

Study ID	Assessment of functional/neurological status	Preop MCS 1 + 2	Preop MCS 3 + 4 + 5	Postop MCS 1+2	Postop MCS 3 + 4 + 5	Preop Frankel D + E	Preop Frankel A + B + C	Postop Frankel D + E	Postop Frankel A + B + C	Worsened neurologic status	Unchanged status	Improved status	Mean length of hospital stay (days)
Champeaux-Depond 2022	Based on predetermined criteria, such as independence at home, hospitalization, and rehabilitation...												8, (range: 7-13)
Kobayashi 2021	Modified McCormick Scale	67	49	96	20					9	34	73	
Pettersson-Segerlind 2021	Modified McCormick Scale<br>American Society of Anesthesiologists (ASA)	80	49							2	66	61	
Wach 2021	Modified McCormick Scale<br>Karnofsky Performance Status (KPS)<br>American Society of Anesthesiologists (ASA) physical status score	83	40	91	31								
Corell 2021	Modified McCormick Scale<br>Karnofsky performance status (KPS)	49	62	74	36					3	48	59	

Baro 2021	Modified McCormick Scale	53	37	75	15					8	29	53	
Kilinc 2021	Eastern Cooperative Oncology Group (ECOG) performance status<br>ASA (American Society of Anesthesiologists) Physical Classification									9	41	72	7.7
Tominaga 2021	Japanese Orthopedic Association score (JOA)									0	0	29	
Davarski 2021	Modified McCormick Scale<br>Medical Research Council (MRC) grading system	2	29	15	15					4	0	26	14.5, (range: 9-30)
Kobayashi 2021	Modified McCormick Scale<br>ASIA scale	30	23	43	10					1	12	40	
Ono K. 2021	Unclear									0	0	21	
Ampie 2021	American Society of Anesthesiologists (ASA) physical status score									6	9	30	Median: 4
Krauss 2021	Unclear									5		10	
Kwee 2020	Modified Rankin Scale (mRS)<br>Karnofsky Performance Score (KPS)									19	24	117	12.3 ± 8.2, (range: 2–55)

Hohenberger 2020	Japanese Orthopedic Association score (JOA)									1	31	13	
Han 2020	Modified McCormick Scale	12	6							2	3	14	
Naito 2020	Modified McCormick Scale<br>Sensory pain scale												
Voldřich 2020	Modified McCormick Scale	44	40	70	14					2	39	53	
Xu 2019	ASIA scale<br>Visual Analogue Scale (VAS) for pain									0	2	15	6.8,<br>(range 5-10)
Elkatatny 2019	Medical Research Council (MRC) grading system												
Noh 2019	Nurick grading scheme									0	2	11	
Hua 2018	McCormick Scale	129	65	158	36					19	95	80	
Schwake 2018	Modified McCormick Scale<br>Karnofsky performance status (KPS)	50	38	73	12					2	25	58	
Gilard 2018	McCormick Scale<br>Karnofsky Performance Score (KPS)	56	31	76	11					4	18	65	
Santos 2018	McCormick Scale	29	22	42	8					2	11	37	
Onken 2018	Modified McCormick Scale<br>Karnofsky Performance Score (KPS)									6	118		9

Raco 2017	Modified McCormick Scale<br>Frankel Scale	43	130	120	53	58	115	124	49	12	11	150	
Notani 2017	Nurick grading scheme									0	0	12	
Davies 2017	Nurick grading scheme									3	0	25	
Wu 2017	Modified McCormick Scale	8	6	12	2					2	0	12	
Zham 2016	Frankel Scale					31	8	28	11	7	32		
Maiti 2016	Modified McCormick Scale	0	38	29	9								
Pompili 2016	Modified McCormick Scale<br>Karnofsky Performance Score (KPS)<br>Dennis Pain Scale												8, (range: 5-19; for both spinal meningiomas and schwannomas).
Tola 2016	McCormick Scale<br>Visual analogue scale (VAS) for pain	7	13	12	8					0	8	12	7 ± 2
Kim 2016	Modified McCormick Scale	26	15	36	5					0	4	16	≈ 7
Lonjon 2016	McCormick Scale<br>Karnofsky Performance Score (KPS)<br>Nurick Scale	14	8							3	6	14	18 ± 16, (range: 4-58)
Ye 2016	McCormick Scale<br>Frankel scale	18	7			20	5						
Haq 2015	Unclear									2	9	37	
Sun 2015	McCormick Scale	1	19	19	1					1	0	19	

Golanov 2015	Frankel Scale<br>Karnofsky Performance Status (KPS)<br>Visual Analogue Scale (VAS) for pain					16	2	16	2	0	17	1	
Arima 2014	Modified McCormick Scale<br>Sensory pain scale									1	8	14	
Tsuda 2014	Unclear									2	0	12	
Riad 2013	McCormick Scale	11	4	14	1					0	2	13	
Kufeld 2012	Arbitrary score for neurological deficits<br>Visual analogue scale (VAS) for pain												
Iacoangeli 2012	Unclear									3		62	5.83,<br>(range: 4-10)
Postalci 2011	Frankel Scale					38	8	39	7	4	14	28	
Sandalcioglu 2008	Frankel Scale					80	51	114	17	5	126		
Boström 2008	Frankel Scale					46	15	60	1	0	33	29	
Schröder 2008	Neurological Scoring System according to Klekamp & Samii									1	7	22	
Setzer 2007	McCormick Scale	58	22	65	15					5	57	18	
Yoon 2007	Unclear									2	6	30	

Schaller 2005	Medical Research Council (MRC) grading system									1	6	19	
Peker 2005	Frankel Scale					35	6	11	30				
Haegelen 2005	Levy score									5	0	28	
Morandi 2004	Solero Score<br>American Society of Anesthesiologists (ASA) physical status score									0	0	30	
Cohen-Gadol 2003	Nurick grading scheme												
Gambardella 2003	McCormick scale	6	4	10	0					0	2	8	
Gezen 2000	Unclear									1	5	35	

MCS = McCormick Scale

Table S13. Markers of neurologic outcomes as described by the included studies

Study ID	Markers of favorable neurological outcomes or postoperative improvement (p-value)	Markers of unfavorable neurological outcomes (p-value)	Markers of postoperative deterioration (p-value)
Champeaux-Depond 2022	- Younger age **(p<0.001)**<br>- Sensory deficits as a presenting symptom **(p=0.006)**<br>- Surgical approach (p=NS)	- Worse preoperative functional status **(p<0.001)**<br>- Care-provider dependent patient **(p<0.001)**<br>- Older age **(p<0.001)**<br>- Motor deficits, bladder or gait dysfunction **(p<0.001, p<0.001 and p=0.048)**<br>- Higher WHO grade **(p=0.023)**<br>- Higher mortality-morbidity index **(p<0.001)**<br>- Surgical delay more than 30 or 90 days (**p=0.009** and p=NS)<br>- Male sex (p=NS)	
Kobayashi 2021	Absence of calcification **(p<0.05)**		
Tominaga 2021	- Simpson grade 1 resection (vs. dura preservation technique) (p=NS)	- Simpson grade 1 resection (vs. dura preservation technique) (p=NS)	
Pettersson-Segerlind 2021	- Larger tumor area **(p=0.03)**<br>- Greater spinal cord compression **(p<0.001)**		- Longer wait time before elective surgery **(p=0.005)**<br>- Older age (p=NS)<br>- Male sex (p=NS)<br>- Higher ASA class (p=NS)<br>- Craniocaudal location (cervical) (p=NS)<br>- Ventral attachment (p=NS)<br>- MIB-index (p=NS)<br>- Higher Simpson grade (p=NS)

Corell 2021	- Greater tumor occupancy (>65% vs. ≤65%) **(p<0.01)**<br>- Less spinal cord compression (p=NS)		- Smaller tumor occupancy (p=NS)<br>- Greater spinal cord compression (p=NS)
Baro 2021		- Greater tumor occupancy **(p=0.005)**<br>- Higher intensity preoperative T2 cord signal changes **(p<0.05)**<br>- Worse preoperative neurological function (p=NS)<br>- Poor postoperative cord re-expansion (p=NS)	
Kilinc 2021		- Obesity **(p=0.05)**<br>- Simpson grade ≥IV **(p<0.001)**<br>- ASA class **(p=0.002)**<br>- Craniocaudal location (lumbar) **(p<0.002)**<br>- Previous surgery **(p=0.01)**<br>- Revision surgery **(p=0.03)**<br>- Male sex **(p=0.03)**<br>- Older Age **(p=0.002)**<br>- Tumor recurrence **(p=0.05)**	
Davarski 2021		- Worse neurological function **(p=0.026)**<br>- Presence of bladder and/or bowel dysfunction **(p=0.009)**<br>- Higher age (p=NS)<br>- Duration of symptoms (p=NS)<br>- Craniocaudal tumor location (p=NS)<br>- Ventral duraL attachment (p=NS)<br>- Number of segments involved (p=NS)<br>- Presence of calcification (p=NS)<br>- Higher WHO grade (p=NS)	
Kobayashi 2021			- Longer duration of symptoms **(p=0.041)**<br>- Worse preoperative functional function **(p=0.024)**

			- Older age (>50 y. vs. <50 y.) (p=NS)<br>- Male sex (p=NS)<br>- Craniocaudal tumor location (p=NS)<br>- Axial tumor location (p=NS)<br>- Presence/Abscence of dural tail (p=NS)<br>- Higher WHO grade (p=NS)<br>- Higher Simpson grade (p=NS)
Wach 2021		- Older age **(p<0.001)**<br>- Longer symptom duration **(p=0.005)**<br>- Presence of myelomalacia **(p<0.001)**<br>- Presence of dural tail sign **(p=0.02)**<br>- Number of segments involved (p=NS)<br>- Ventral dural attachment (p=NS)<br>- Higher Simpson grade (p=NS)<br>- Higher WHO grade (p=NS)<br>- Higher MIB-index (p=NS)	- Older age **(p=0.027)**
Kwee 2020	- Positive history of cardiovascular disease **(p=0.017)**<br>- Surgery after 2009 (the year of the introduction of intraoperative monitoring) **(p=0.037)**<br>- Younger age (p=NS)<br>- Better preoperative neurological function (p=NS)<br>- Surgical treatment (vs. observation alone) (p=NS)	- Male sex **(p=0.003)**<br>- Higher WHO grade (2) **(p=0.013)**<br>- Presenting symptoms (p=NS)<br>- Perioperative complication (p=NS)<br>- Greater tumor size (p=NS)<br>- Positive history of malignancy (p=NS)	
Hohenberger 2020		- Use of intraoperative monitoring (p=NS)	
Hua 2018		- Recurrent tumor **(p=0.006)**<br>- Higher WHO grade **(p<0.001)**<br>- Worse preoperative neurological functions **(p<0.001)**<br>- More segments involved **(p=0.034)**	- Recurrent tumor **(p<0.001)**<br>- Worse preoperative neurological functions **(p<0.001)**<br>- Higher tumor grade **(p=0.001)**<br>- Higher tumor Ki-67 index **(p<0.001)**

		- Higher Ki-67 index **(p<0.001)**<br>- Older age (>60 y. vs. <60 y.) (p=NS)<br>- Male sex (p=NS)<br>- Craniocaudal tumor location (p=NS)<br>- Ventral tumor attachment (p=NS)<br>- Symptom duration (<8 mos vs. ≥8mos) (p=NS)<br>- Simpson grading (p=NS)<br>- Progesterone receptor (PR) status (p=NS)	- Older age (>60 y. vs. <60 y.) (p=NS)<br>- Male sex (p=NS)<br>- Craniocaudal tumor location (p=NS)<br>- Ventral dural attachment (p=NS)<br>- More segments involved (p=NS)<br>- Symptom duration (<8 mos vs. ≥8mos) (p=NS)<br>- Simpson grading (p=NS)<br>- Progesterone receptor (PR) status (p=NS)
Schwake 2018		- Older age **(p=0.001)**<br>- Worse preoperative neurological function **(p=0.001)**	- Surgical approach (p=NS)
Gilard 2018			- Ventral dural attchment **(p=0.03)**<br>- Better preoperative neurological function (on both MMS and KPS) **(p=0.04)**<br>- Higher WHO grade **(p<0.01)**<br>- Male sex (p=NS)<br>- Older age (p=NS)<br>- Clinical presentation (p=NS)<br>- Longer symptom duration (p=NS)
Raco 2017	- Paresthesia as a presenting symptom **(p=0.025)**<br>- Tumors with no anterior attachments **(p=0.016)**<br>- Year operated (1992-2011 vs. 1976-1991) (p=NS)	- Ventral dural attachment **(p=0.21)**<br>- Sphincter disturbance as a presenting symptom **(p=0.006)**<br>- Higher Simpson grade (II and III) **(p=0.04)**<br>- Worse preoperative neurological function **(p=0.01)**<br>- Ventral (AND) recurrent tumors **(p=0.003)**<br>- Higher SPES **(p=0.001)**<br>- Dumbbell tumor (p=NS)	- Operation of recurrent lesions **(p=0.003)**<br>- Longer duration of symptoms **(p=0.05)**<br>- Perioperative complications (p=NS)

Zham 2016		- Craniocaudal location (cervical) **(p=0.027)**<br>- Adhesion during surgery and incomplete removal of tumor **(p=0.012)**<br>- Psammomatous histological subtype **(p=0.003)**<br>- Higher WHO grade **(p=0.026)**<br>- Tumor size (p=NS)<br>- Older age (p=NS)<br>- Male sex (p=NS)	
Maiti 2016		- Ventral dural attachment **(p=0.003)**<br>- Greater tumor size (occupying ≥75% vs. <75% of the spinal canal) **(p=0.02)**<br>- Presence of T2 signal intensity changes of spinal cord **(p=0.022)**<br>- Worse preoperative neurological functions **(p=0.003)**<br>- Male sex (p=NS)<br>- Older age (<50 y. vs. >50 y.) (p=NS)<br>- Race (Caucasian vs African American) (p=NS)<br>- Association with NF2 (p=NS)<br>- Higher WHO grade (p=NS)<br>- Craniocaudal location (p=NS)<br>- Number of segments involved (≤2 vs. >3) (p=NS)<br>- Presence of dural tail (p=NS)	
Sandalcioglu 2008			- Calcification **(p<0.0001)**<br>- Ventral dural attachment (p=NS)<br>- Male sex (p=NS)
Yoon 2007		- Craniocaudal tumor location (p=NS)	

Setzer 2007		- En plaque growth **(p<0.03)**<br>- Higher Simpson grade **(p<0.006)**<br>- Worse preoperative neurological function **(p<0.006)**<br>- Higher WHO grade **(p<0.012)**<br>- Invasion of the arachnoid/pia mater **(p<0.03)**	- Invasion of the arachnoid/pia mater **(p<0.03)**<br>- Longer duration of symptoms **(p<0.001)**
Schaller 2005	- Histological subtype (not psammomatous vs. psammomatous) **(p<0.05)**<br>- Smaller tumor size (p=NS)<br>- Female sex (p=NS)		
Morandi 2004		- Worse preoperative neurological function (p=NS)	

The p-value of significant correlations were marked in bold character, NS = Not Significant, WHO = World Health Organization

Table S14. Recurrence rate and markers of recurrence

Study ID	Number of tumors operated	Mean follow-up time (mos)	Number of recurrences	Recurrence rate	Mean time to recurrence	Range of recurrence time	Recurrence markers (p-value)
Saiwai 2021	38	121.5 ± 9.0	3	7.89%	17.6 ± 51.2	59–153	- Dura preservation technique (Saito method) vs. Simpson grade 2 (p=NS)
Wach 2021	80	28.8	2	2.50%	18	12-24	- Higher MIB-1 labeling index (≥5% vs. <5%) **(p<0.05)**
Tominaga 2021	29	Median: 132, (IQR: 120–160.5)	3	10.34%	median: 95		- Higher Simpson grade **(p<0.05)**
Pettersson-Segerlind 2021	129	98.4	6	4.65%	48.9 ± 30	4-85.2	- Higher WHO grade (p=NS)<br>- Younger age (p=NS)
Kobayashi 2021	116	84.8 ± 52.7	8	6.90%	72.9	24-115	- Male sex **(p=0.018)**<br>- Presence of dural tail **(p=0.046)**<br>- Higher Simpson grade **(p< 0.01)**<br>- Younger age (p=NS)<br>- Craniocaudal tumor location (p=NS)<br>- Axial tumor location (p=NS)<br>- Longer duration of symptoms (p=NS)<br>- Worse preoperative functional scores (p=NS)<br>- Higher WHO grade (p=NS)
Krauss 2021	17	95.3	8	47.06%	37.1 ± 24	12-78	- Male sex **(p=0.03)**<br>- Craniocaudal tumor location (not thoracic vs thoracic) **(p=0.001)**<br>- Younger age (p=NS)<br>- Larger tumor size (p=NS)<br>- Pain at presentation (p=NS)<br>- Sensory deficits at presentation (p=NS)<br>- Motor deficits at presentation (p=NS)<br>- Bladder and bowel dysfunction at presentation

							(p=NS)<br>- Higher Simpson grade (p=NS)
Kilinc 2021	119	25.4 ± 37.1	9	7.56%	120.1	12–348	- Presence of calcification **(p=0.006)**<br>- Simpson grade ≥ 4 **(p<0.001)**<br>- Ventral dural attachment<br>- Craniocaudal location (p=NS)<br>- Number of spinal segments involved (p=NS)<br>- Obesity (p=NS)<br>- Diabetes (p=NS)<br>- Hypertension (p=NS)<br>- Surgeon experience (p=NS)
Ampie 2021	46	Median: 53	1	2.17%	1	n/a	
Davarski 2021	31	43	1	3.23%	48	n/a	
Corell 2021	111	107 ± 108	3	2.70%	71.7 ± 60	11-131	
Baro 2021	90	Median: 19	2	2.22%			
Aoyama 2021	15	> 60	0	0.00%			
Maiuri 2020	56	median: 192	6	10.71%			- Higher MIB-1 labeling index **(p=0.0001)**<br>- Arachnoid invasion **(p=0.04)**<br>- Simpson grade 1 vs. 2 (p=NS)<br>- Younger age (p=NS)<br>- Male sex (p=NS)<br>- Craniocaudal location (p=NS)<br>- Degree of progesterone or estrogen receptor positivity (p=NS)<br>- Vascularity and consistency of the tumor (p=NS)
Han 2020	20	79.6 ± 39.9	7	35.00%	80.9 ± 69.7	15-108	- Higher WHO grade was associated with a shorter duration until recurrence **(p<0.01)**
Kwee 2020	159	Median: 9.24	12	7.55%	62.4 ± 52.8		- Simpson grade 4 resection **(p=0.008)**<br>- Younger age **(p=0.006)**

							- Bladder or bowel dysfunction as a presenting symptom **(p=0.029)**<br>- Higher WHO grade (2) (p=NS)<br>- Craniocaudal location (p=NS)
Voldřich 2020	92	32 ± 44.1	4	4.35%	106.5 ± 35.5	78-156	
Naito 2020	70	61	2	2.86%			
Hohenberger 2020	45	34	0	0.00%			
Yeo 2019	105	28	1	0.95%	56	n/a	
Noh 2019	13	68.94 ± 72.14	0	0.00%			
Xu 2019	17	19.8	0	0.00%			
Hua 2018	194	94.34 ± 29.49	9	4.64%	36.22 ± 16.01	15–60	- Male sex **(p< 0.001)**<br>- Higher Simpson grade **(p< 0.001)**<br>- Higher WHO grade **(p< 0.001)**<br>- Recurrent tumors **(p< 0.001)**<br>- Age (<60 y. vs. >60 y.) (p=NS)<br>- Simpson grade 2 vs 1 (p=NS)<br>- Craniocaudal tumor location (p=NS)<br>- Number of segments involved (1 vs. ≥2) (p=NS)<br>- Axial tumor location (p=NS)<br>- Worse preoprative functional scores (p=NS)<br>- MIB index (<3 vs. ≥3) (p=NS)<br>- Progesterone receptor (PR) status (p=NS)
Schwake 2018	84	19 ± 5.16	1	1.19%	40	n/a	
Onken 2018	207	14	10	4.83%	Median: 156		
Gilard 2018	87	92.4 ± 51.9	6	6.90%			
Raco 2017	173	50.8 ± 9.3	4	2.31%	30 ± 36.2	1-76	- Higher Simpson grade **(p=0.043)**<br>- Higher WHO grade **(p=0.002)**

Notani 2017	12	55.4	1	8.33%	132	n/a	
Wu 2017	14	66.1	4	28.57%	25 ± 23	12-60	
Maiti 2016	37	51.2 ± 22.4	4	10.81%	39.75 ± 18.6	19-64	- Male sex **(p<0.001)**<br>- Presence of dural tail **(p=0.04)**<br>- Age (<50 y. vs. >50 y.) (p=NS)<br>- Race (Caucasian vs African American) (p=NS)<br>- Association w/ NF2 (p=NS)<br>- Higher WHO grade (p=NS)<br>- Craniocaudal location (p=NS)<br>- Number of segments involved (≤2 vs. >3) (p=NS)<br>- Ventral dural attachment (p=NS)<br>- Tumor size (occupying ≥75% vs. <75% of the spinal canal) (p=NS)<br>- T2 signal intensity changes of spinal cord (p=NS)
Ye 2016	25	50.6 ± 38.2	13	52.00%	23.5 ± 22	3-65	- Ventral dural attachment **(p=0.012)**<br>- Number of involved segments **(p=0.002)**<br>- Higher Simpson grade **(p=0.034)**<br>- Male sex (p=NS)<br>- Younger age (p=NS)<br>- Longer duration of symptoms (p=NS)<br>- Worse preoperative neurological function (p=NS)<br>- Craniocaudal tumor location (p=NS)<br>- Tumor site (intradural vs. extradural) (p=NS)<br>- Larger tumor size (p=NS)<br>- Higher WHO grade (II vs. III) (p=NS)<br>- Presence of cord invasion (p=NS)<br>- Presence of osteolytic destruction (p=NS)<br>- Presence of calcification (p=NS)
Pompili 2016	29	72	1	3.45%	48	n/a	
Kim 2016	73	111.5	9	12.33%	82 ± 70	3.6-196.2	

Tola 2016	20	40 ± 32	0	0.00%			
Lonjon 2016	23	40 ± 26.5	4	17.39%			
Sun 2015	20	median: 34	1	5.00%	74	n/a	- Higher Simpson grade **(p=0.025)**
Aboul-Enein 2015	16	51.6	4	25.00%	56 ± 18	36-72	
Haq 2015	48	24	6	12.50%			
Tsuda 2014	13	124.8	3	23.08%	12.1		
Arima 2014	23	32.1	3	13.04%			
Riad 2013	15	99	1	6.67%	96	n/a	
Nakamura 2012	68	145.2	12	17.65%	104 ± 68	18-252	- Younger age **(p<0.05)**<br>- Higher Simpson grade **(p<0.0001)**<br>- DIfferent histologic subtypes (p=NS)
Barresi 2012	58	78% of patients had a follow-up of at least 60 months	1	1.72%	53	n/a	
Wang 2012	10	43	7	70.00%	14 ± 10	3-34	
Postalci 2011	35	60	8	22.86%			
Voulgaris 2010	10	26	0	0.00%			
Sacko 2009	102	Median: 49.5	1	0.98%	48	n/a	
Sandalcioglu 2008	131	61	4	3.05%	76.5	36-116	- Ventral dural attachment (p=NS)
Schröder 2008	30	36	3	10.00%	33 ± 29	3-60	
Boström 2008	61	31.3	5	8.20%	36 ± 19	12-60	
Setzer 2007	80	43.5 ± 24.8	8	10.00%	56.6	9.5-132	- Higher Simpson resection grade **(p<0.03)**<br>- Invasion of arachnoid/pia mater **(p<0.001)**

							- Histological tumor type **(p<0.008)**<br>- Higher WHO grade **(p<0.001)**<br>- Younger patient age **(p<0.006)**
Yoon 2007	38	78.8	6	15.79%	100	12-204	
Schaller 2005	33	96 ± 48	1	3.03%	96	n/a	
Haegelen 2005	33	70.7	0	0.00%			
Peker 2005	41	23.2	0	0.00%			
Morandi 2004	30	62.7	0	0.00%			
Cohen-Gadol 2003	80	85	11	13.75%			- Younger age **(p<0.05)**
Gambardella 2003	10	41	0	0.00%			
Arslantas 2003	16	28.8	0	0.00%			
Schick 2001	81	51.6	7	8.64%	63	17-164	
Gezen 2000	36	108	2	5.56%	78	60-96	

The p-value of significant correlations were marked in bold character, NS = Not Significant, WHO = World Health Organization

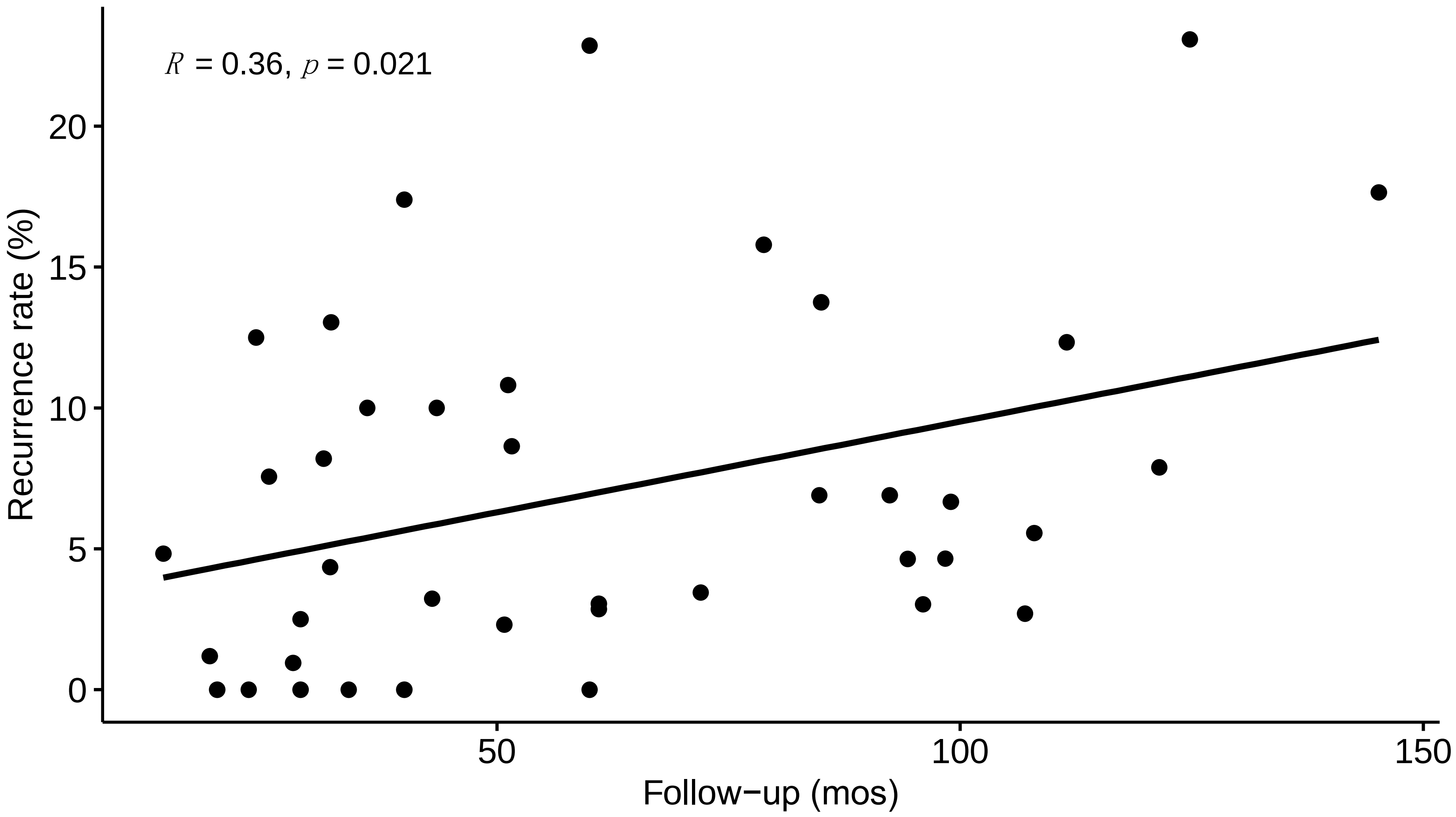

Figure S1. Association between the recurrence rate and the follow-up duration, with the associated Pearson correlation coefficient R and p-value.

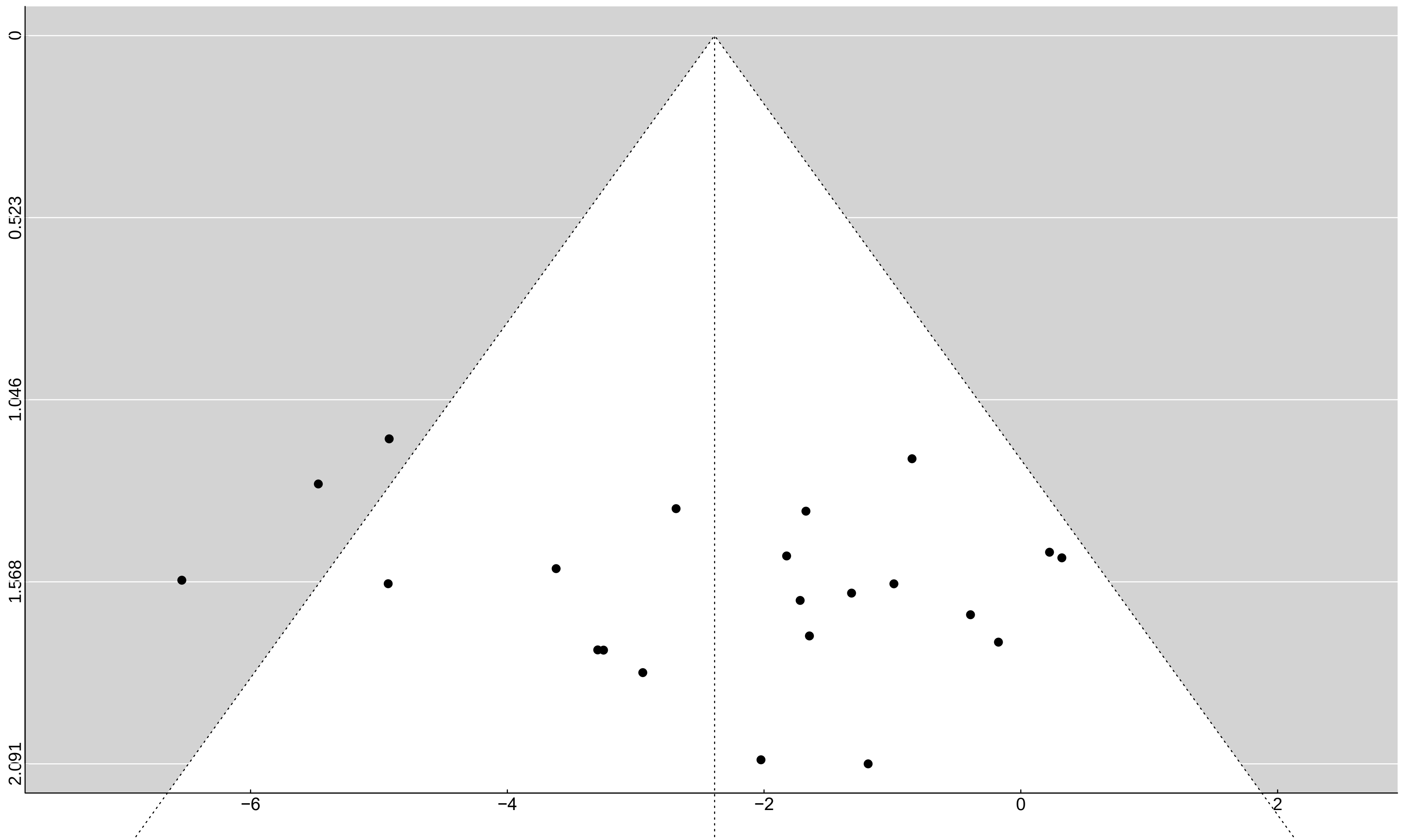

Figure S2. Funnel plot showing the distribution of studies comparing the recurrence rate among low vs. high WHO grade spinal meningiomas.

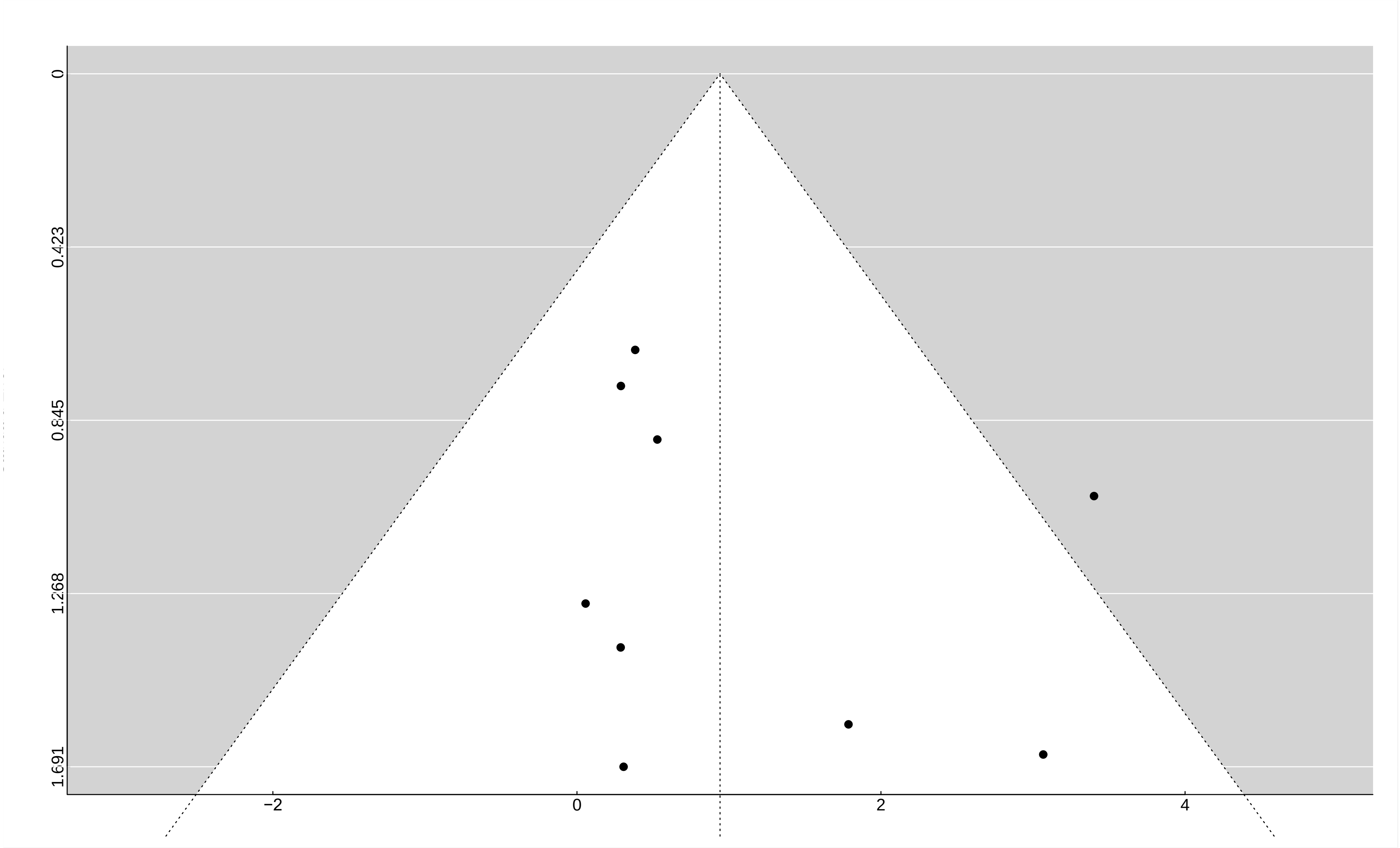

Figure S3. Funnel plot showing the distribution of studies comparing the recurrence rate among ventral vs. non-ventral spinal meningiomas.

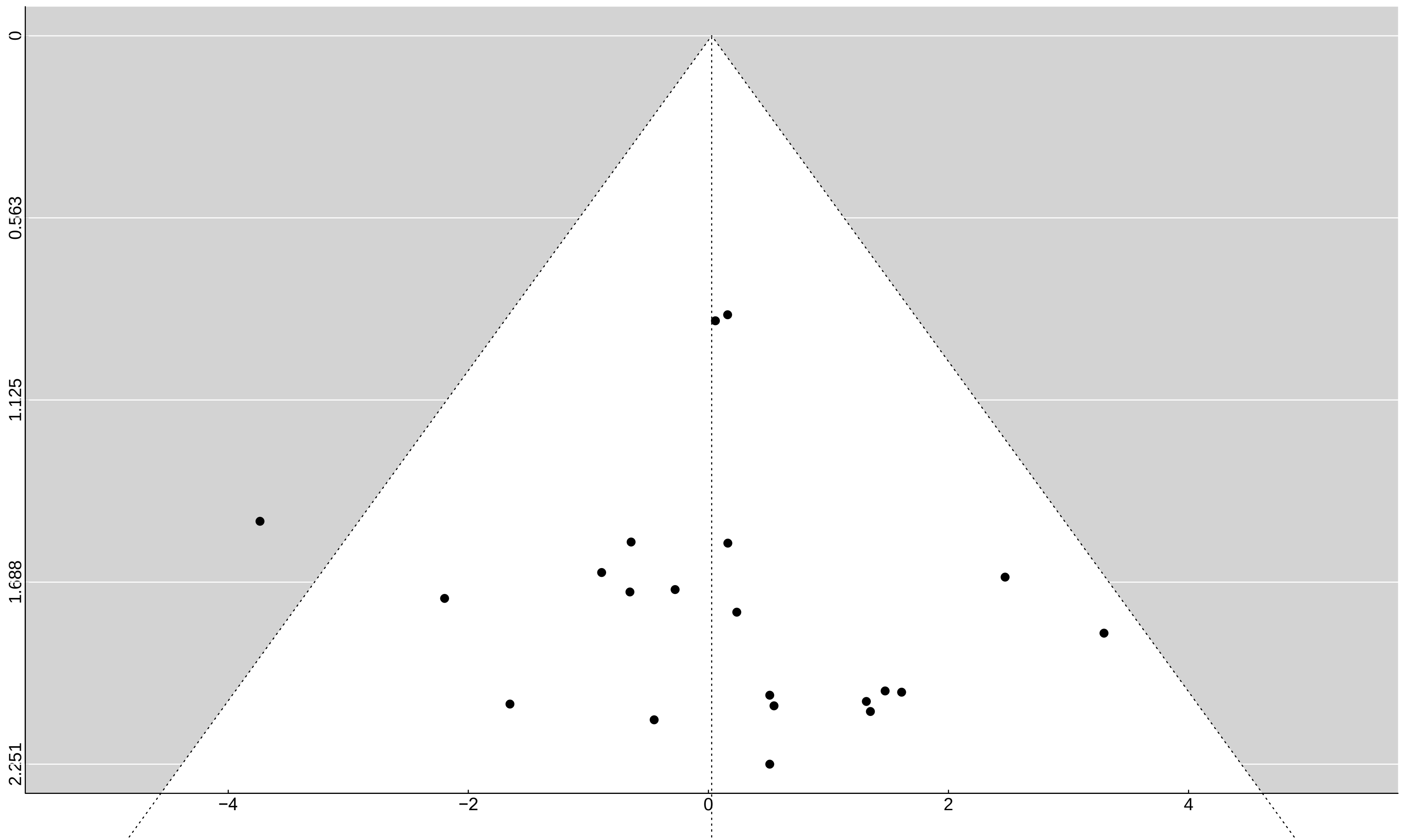

Figure S4. Funnel plot showing the distribution of studies comparing the recurrence rate spinal meningiomas operated with Simpson grade 1 vs. grade 2 resection.

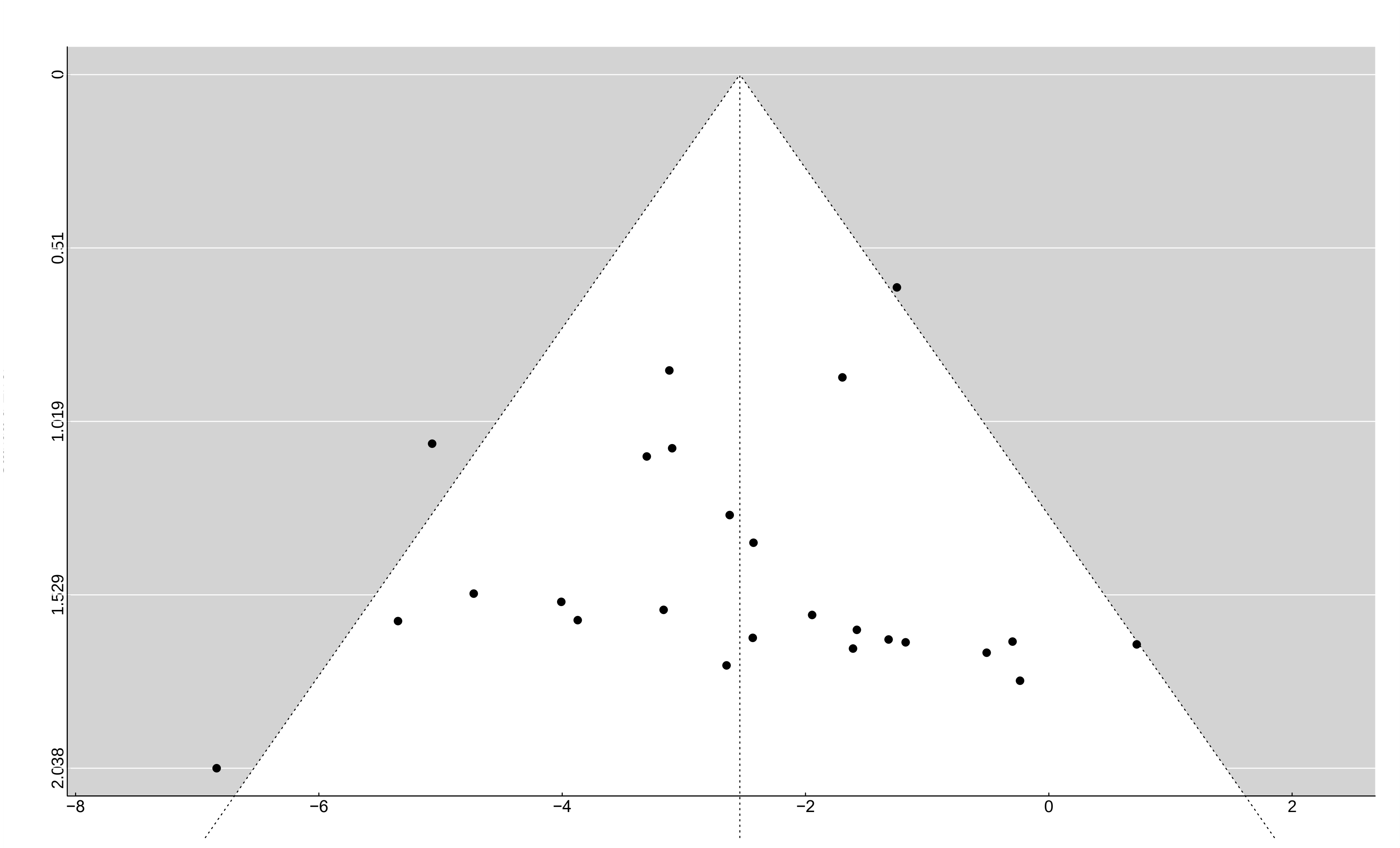

Figure S5. Funnel plot showing the distribution of studies comparing the recurrence rate spinal meningiomas operated with Simpson grade 1 and 2 vs. grade 3, 4, and 5 resections.
